# Calcitonin Gene-Related Peptide Monoclonal Antibodies: Key Lessons from Real-World Evidence

**DOI:** 10.3390/brainsci14090948

**Published:** 2024-09-22

**Authors:** Bianca Orlando, Gabriella Egeo, Cinzia Aurilia, Giulia Fiorentini, Piero Barbanti

**Affiliations:** 1 Headache and Pain Unit, IRCCS San Raffaele, Via della Pisana 235, 00163 Rome, Italy; bianca.orlando@sanraffaele.it (B.O.); gabriella.egeo@sanraffaele.it (G.E.); cinzia.aurilia@sanraffaele.it (C.A.); giulia.fiorentini@uniroma5.it (G.F.); 2 San Raffaele University, 00166 Rome, Italy

**Keywords:** migraine, anti-CGRP mAbs, treatment, real-life, disability

## Abstract

Background: The advent of monoclonal antibodies (mAbs) targeting the calcitonin gene-related peptide (CGRP) pathway has transformed the management of migraine, offering newfound optimism for clinicians and individuals with episodic migraine (EM) and chronic migraine (CM). While randomized controlled trials (RCTs) have provided crucial insights into the effectiveness and safety profiles of these treatments, their translation into real-world clinical practice remains a challenge. Objective: This review aims to conduct a comprehensive assessment of real-world studies, offering valuable insights tailored for practical application in clinical settings. Methods: We conducted a comprehensive literature search in PubMed, SCOPUS, and MEDLINE for real-life studies on erenumab, fremanezumab, and galcanezumab. Abstracts underwent rigorous screening by two reviewers for relevance. Data extraction from selected articles was performed using a standardized form, with verification by a second reviewer. Data synthesis was narrative, following PRISMA guidelines. Results: Our search included 61 pertinent studies conducted between 2019 and 1 March 2024. Real-world study designs demonstrated notable variability in the selection and inclusion of migraine patients, influenced by factors such as attack frequency, data collection criteria, and primary/secondary objectives. Key findings commonly reported considerable improvements in efficacy outcomes (migraine frequency, analgesic use, pain severity, and disability), high responder rates, and optimal safety and tolerability profiles. Conclusions: Real-world evidence underscores the role of anti-CGRP mAbs as targeted therapies for both CM and EM patients. The overall results indicate that the effectiveness and tolerability of anti-CGRP mAbs in real-world applications may exceed those observed in RCTs, an extraordinary finding in clinical neurology.

## 1. Introduction

The introduction of monoclonal antibodies (mAbs) targeting the calcitonin gene-related peptide (CGRP) pathway has revolutionized migraine management thanks to their remarkable efficacy–tolerability profile. This advancement provides renewed optimism for individuals afflicted by migraine, whether episodic (EM) or chronic (CM), representing a valuable alternative therapeutic option for patients who failed traditional preventive treatment [1].

Erenumab was the first anti-CGRP mAb approved for the preventive treatment of migraine, functioning through the selective inhibition of the CGRP receptor. In contrast, galcanezumab, fremanezumab, and eptinezumab prevent the biological activity of CGRP by targeting the ligand itself [2].

Since 2017, randomized controlled trials (RCTs) have elucidated the efficacy and safety of anti-CGRP mAbs in EM—with or without aura—or CM [2]. While RCTs offer strong evidence of the efficacy of anti-CGRP mAbs in controlled settings, a gap between these trials and real-world clinical practice often emerges. This discrepancy is largely attributed to the differences in patient demographics included in RCTs, who may not accurately represent the broader, more diverse patient population seen in everyday clinical settings, particularly in terms of age, comorbidities, and disease severity [3].

The expanding global accessibility of real-world evidence (RWE) observational studies has furnished more extensive information compared to that offered by RCTs in addressing the ongoing challenge of therapeutic decision-making. The importance of RWE studies is diverse and is anticipated to have a growing impact on regulatory and reimbursement decisions. These studies capture a broader, more varied, and complex array of patient cohorts and demographic groups often not included in initial RCTs. Further, they provide longer follow-up periods for evaluating drug safety and effectiveness and act as crucial resources for generating hypotheses on predictors of response and for assessing patient-reported outcomes [4,5,6,7].

This review offers a thorough examination of RWE studies evaluating the use of erenumab, fremanezumab, and galcanezumab in the prevention of migraine, enhancing the understanding gained from RCTs with data derived from actual clinical practice settings.

## 2. Methods

We conducted a comprehensive literature search to identify studies pertaining to erenumab, fremanezumab, and galcanezumab. We did not consider eptinezumab due to its more recent market release. Specifically, we scoured peer-reviewed electronic literature databases such as PubMed, SCOPUS, and MEDLINE for all available publications up to March 2024. The keywords “migraine”, “erenumab”, “fremanezumab”, “galcanezumab” “anti-CGRP mAbs”, “treatment”, “real-life”, and “real-word” were used. We have divided the studies into groups for each mAb to allow detailed discussion of the findings. 

Following the removal of duplicate records, abstracts were meticulously screened. Eligible studies were those conducted in populations diagnosed with migraine with or without aura, containing original data, specifying the type of mAbs utilized, and published in English. Case reports were excluded from consideration. The findings were verified by the second reviewer. Selected abstracts proceeded to full-text review.

The extracted data encompassed various study characteristics, including authorship, publication year, study design, participant demographics, intervention specifics, outcomes assessed, and key findings. We describe the findings, mostly utilizing the most common indicators in clinical practice that demonstrate the effectiveness of mAbs, including the reduction in the number of monthly migraine days (MMD), monthly headache days (MHD), monthly acute migraine medication intake (MAI), pain intensity measured as Numeric Rating Scale (NRS) score, Headache Impact Test-6 score (HIT-6), and Headache Migraine Disability Assessment Score (MIDAS), response rate measured as ≥30%, ≥50%, ≥75%, and 100% reduction in MHD/MMD compared to baseline, and improvements in patient-reported outcomes. Proportions of CM patients who reverted to EM and CM patients who discontinued medication overuse were also evaluated, wherever the data could be extracted. Safety and tolerability were assessed by monitoring adverse events. Synthesis of the extracted data was performed narratively to present a comprehensive overview of anti-CGRP mAbs for migraine prevention. This review adheres to the Preferred Reporting Items for Systematic Reviews and Meta-Analyses (PRISMA) guidelines to ensure transparent reporting of methods and results.

## 3. Results 

### Study Characteristics

**Figure 1:**
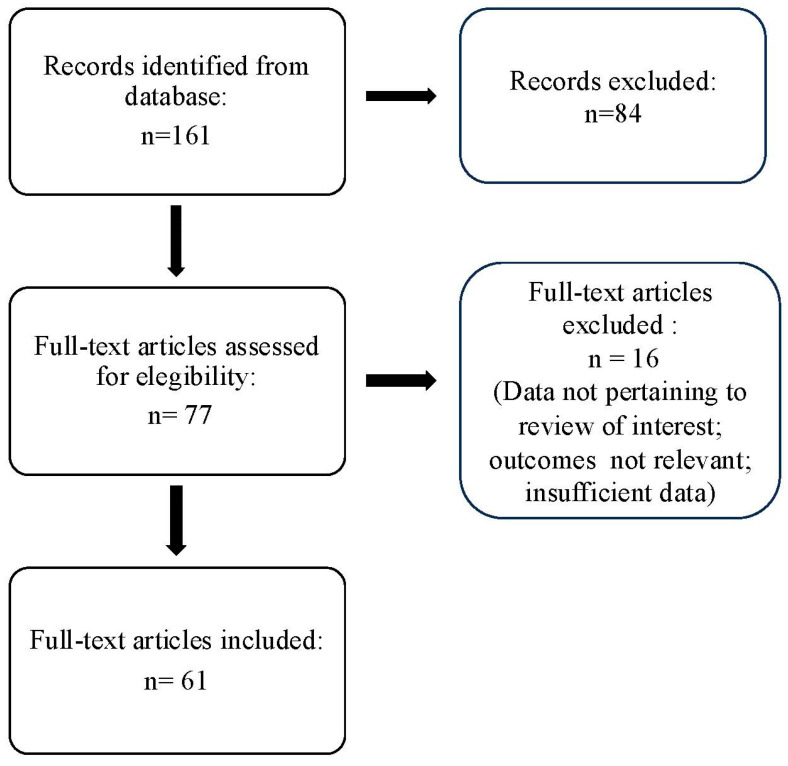
PRISMA flow diagram.

**Figure 2:**
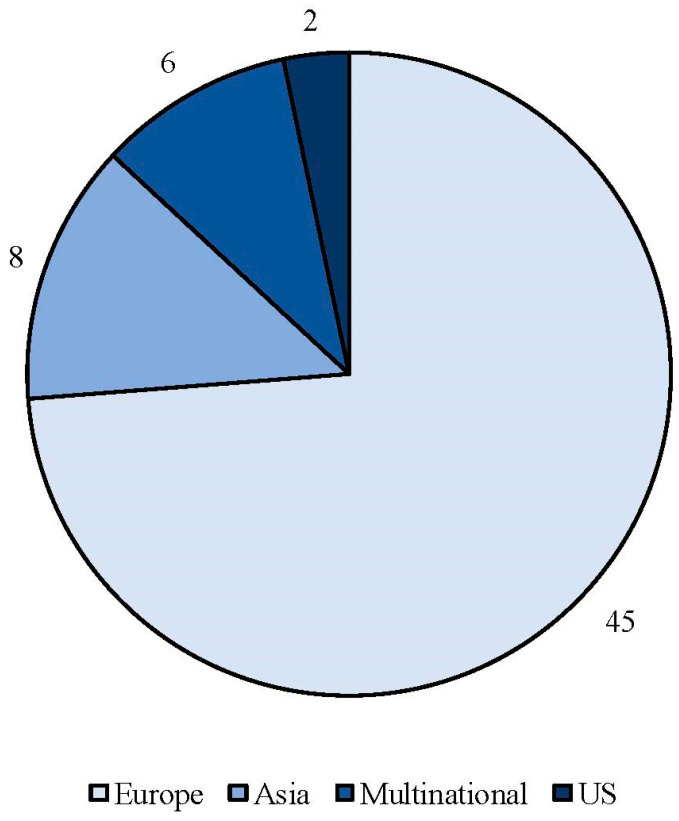
Geographic distribution of countries involved in real-life studies using anti-CGRP mAbs.

**Figure 3:**
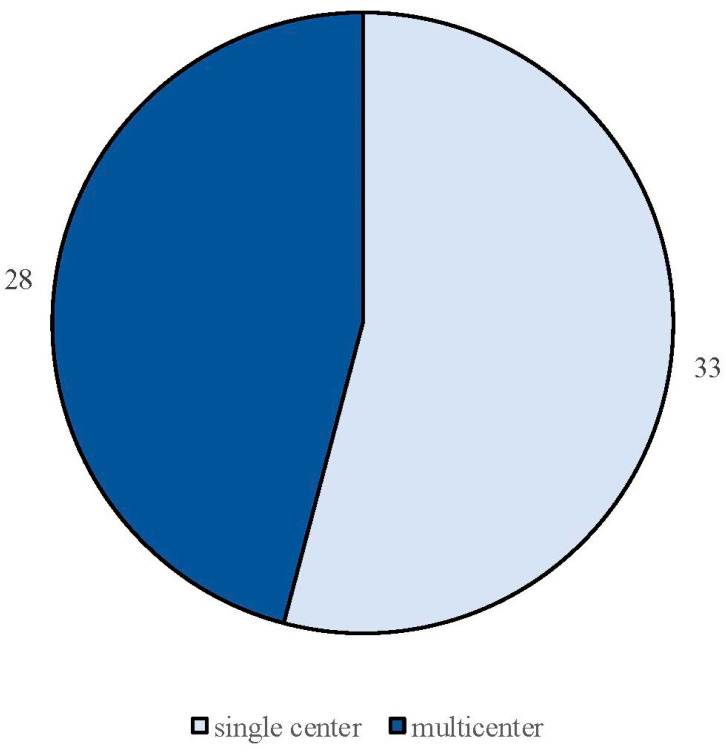
Classification of real-life studies based on their design as either single-center or multicenter studies.

**Figure 4:**
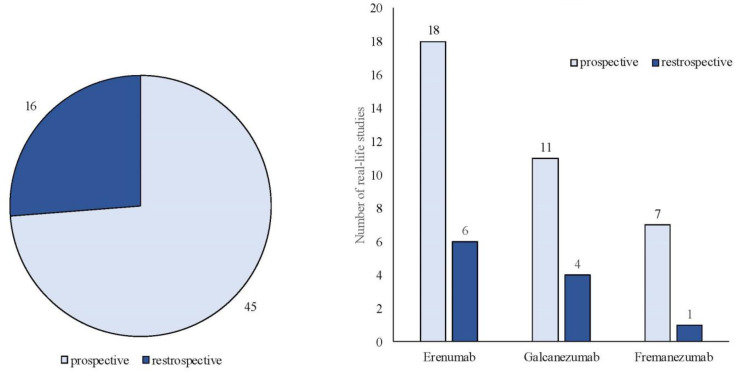
Classification of real-life studies based on their design as either prospective or retrospective studies.

**Figure 5:**
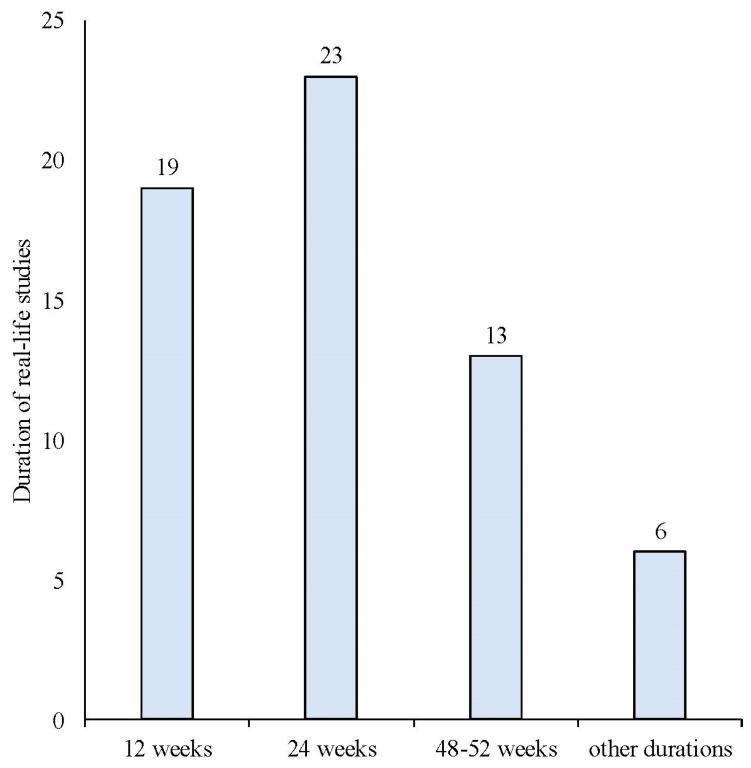
Duration of the real-life studies conducted with anti-CGRP mAbs.

**Figure 6:**
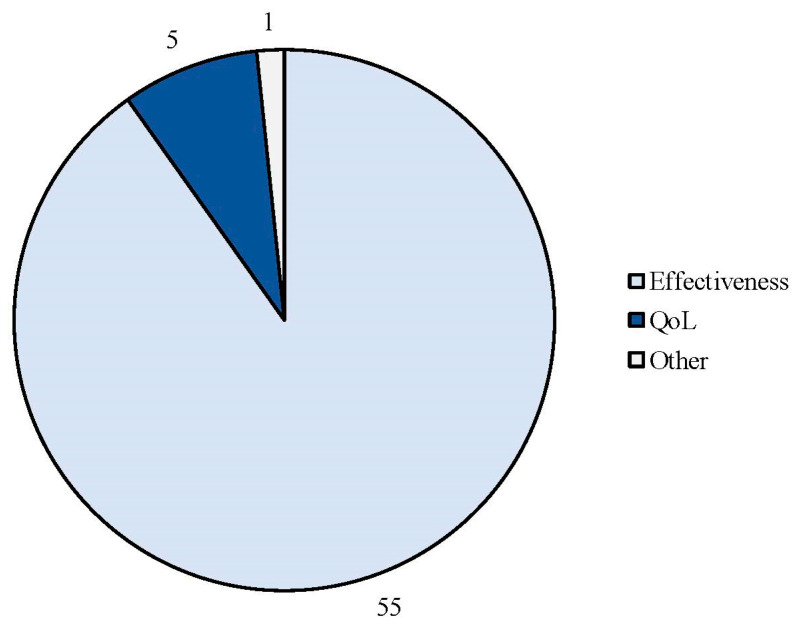
Efficacy endpoints evaluated in the real-life studies involving anti-CGRP mAbs.

**Figure 7:**
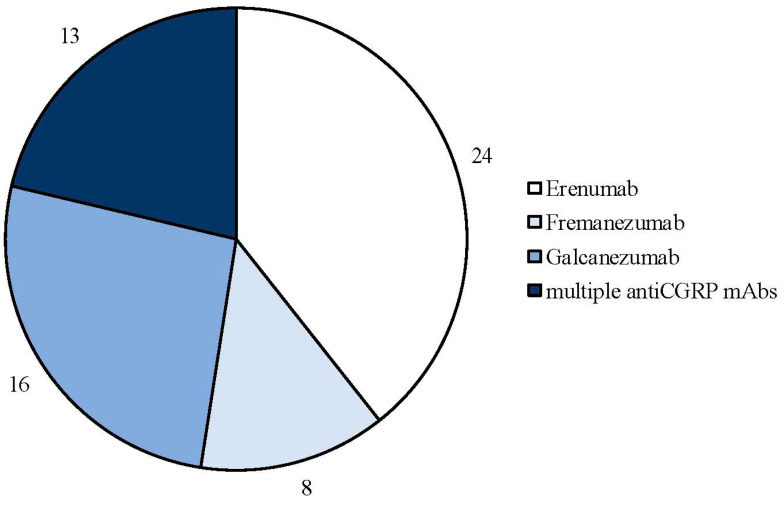
Number of real-life studies conducted with each type of anti-CGRP monoclonal antibody.

The search strategy identified 61 articles that met the criteria for full-text review, covering studies conducted ranging from 2019 to 2024 (Figure 1). Most investigations were carried out in Europe (45/61, 73.7%), followed by Asia (8/61, 13.1%) and the US (2/61, 3.2%), with 9.8% (6/61) being international (Figure 2). Within Europe, 32 studies were conducted in Italy, 6 across the United Kingdom, 4 in Germany, 2 across the Netherlands, Ireland, Spain, Denmark, Czechia, and the Russian Federation, and 1 were conducted in Greece, Switzerland, Poland, England, and France. The majority of the studies (33/61, 50.8%) were single-center, while 47.5% (28/61) were multicenter (Figure 3). The most common study design was prospective cohort studies (45/61, 73.7%) followed by retrospective cohort ones (16/61, 26.2%) (Figure 4). The duration of the studies varied, with 31.2% (19/61) lasting 12 weeks, 37.7% (23/61) lasting 24 weeks, 21.3% (13/61) lasting 48 weeks or more, and 9.8% (6/61) categorized under other durations (Figure 5). The primary endpoint was effectiveness in 90.1% (55/61) of the studies, quality of life/disability in 8% (5/61), and other endpoints in 1.6% (1/61) (Figure 6). Erenumab was the most extensively studied anti-CGRP mAb, followed by galcanezumab and fremanezumab. Several studies considered more than one anti-CGRP mAb (Figure 7) [8,9,10,11,12,13,14,15,16,17,18,19,20,21].

Study design and enrollment inclusion criteria varied across the different real-world studies, reflecting the diversity of clinical practice. Additionally, the studies exhibited heterogeneity in sociodemographic and clinical details, as well as in the primary and secondary efficacy endpoints. Consequently, the following clinical details and their corresponding ranges exclusively pertain to the studies that included these aspects. While most studies provided distinct effectiveness results for patients with EM and those with CM, some studies did not specify outcomes based on migraine frequency.

(a) Erenumab

We identified 24 RWE studies including 7940 patients, predominantly females (82%), diagnosed with either EM (n = 1237) or CM (n = 6703), with a mean age ranging from 43 to 53 years (Table 1) [4], [22,23,24,25,26,27,28,29,30,31,32,33,34,35,36,37,38,39,40,41,42,43,44]. One study specifically focused on patients older than 65 years [40]. Most of these investigations were prospective (n = 19) [4,22,23,25,26,27,29,30,32,33,34,35,36,38,39,40,41,42,43], with six being retrospective (Figure 3) [24,28,31,37,44].

Fifteen were conducted across multiple centers [4,23,25,28,29,30,31,34,37,38,39,41,42,43,44], whilst 9 were single-center studies [22,24,26,27,32,33,35,36,40].

Twenty studies took place within a single country [4,22,23,24,25,26,27,29,30,31,32,33,34,35,36,37,39,40,41,43], while four were international [28,38,42,44]. The duration of these studies varied, with 1 lasting 2 months [22], 8 enduring ≥ 3 months [23,24,28,31,34,36,38,43], 8 extending to 6 months [25,26,27,29,33,37,39,40], 4 for 9–12 months [4,30,35,41], and 3 for more than 12 months [32,42,44].

The duration of the migraine among participants ranged from 5 to 33 years, with baseline values of MMD/MHD spanning from 9.4 to 26, NRS score from 6.8 to 10, HIT-6 from 64.2 to 67.8, and MIDAS from 77.5 to 130.5. At baseline, the MAI ranged from 11.6 to 26.7, with the proportion of subjects experiencing medication overuse varying from 52% to 95%. The average number of prior therapeutic failures spanned from 3.6 to 6.9 (Table 1). Only one study conducted a comparative analysis of outcomes between men and women [28]. 

Between 11% and 74% of patients required dose escalation from 70 mg to 140 mg, with the latter usually being more effective. Changes in MMD, MHD, NRS, MAI, MIDAS, and HIT-6, as well as ≥50%, ≥75%, and 100% response rates, are detailed in Table 2. Three studies reported ≥30% responses [26,27,38]. Treatment with erenumab led to a transition from CM to EM in 22–83% of individuals and from medication overuse to no medication overuse in 25% to 71.9% of patients. Some studies also evaluated patient reported outcomes, assessing aspects such as disability, quality of life, quality of sleep, pain catastrophizing, impact of migraine on partners and adolescent children, treatment satisfaction, and subjective cognitive impairment during migraine attacks [26,39,42].

**Table 1:** Erenumab in RWE studies.

Author/ Year	N° of pts	Observation Period	Study Type/Center/National–International	Primary Endpoint	Secondary Endpoints	Results	Safety Findings
Barbanti et al., 2019 [22]	HFEM/CM: 13/65	8 weeks	P S N = Italy	Change in MMD at weeks 5–8 vs. baseline	Change in MAI, ≥50%, ≥75%, and 100% RR and any variation in VAS and HIT-6 scores.	*Primary endpoints:* HFEM: MMD-7; CM: MH9,7Ds—15. *Secondary endpoints:* HFEM: MAI-7; VAS-7; HIT-6 −30; ≥50% ≥ 75% and 100% R were 100%. CM: MAI −15, VAS −3, and HIT-6 −12.8, ≥50% R 87.5%, ≥75% R 37.5%.	One AE (injection-site erythema) in a single patient (1.3%).
Barbanti et al., 2020 [23]	HFEM/CM: 103/269	12 weeks	P M (n = 9) N = Italy	Change in MMD at weeks 9–12 vs. baseline in HFEM and CM.	Change in MAI, ≥50%, ≥75%, and 100% RR and any variation in VAS and HIT-6 scores.	*Primary endpoints:* HFEM: MMD −4.5; CM: MMD −9.3. *Secondary endpoints:* HFEM: VAS −1.9; HIT −10.7; MAI from 12.0 (IQR 10.0–14.0) to 5.0 (IQR 3.0–7.0); RR: ≥50% 59.4%; ≥75% 16.8% and 100% 1. CM: VAS −1.7 ± 2.0; HIT −9.7; MAI from 20.0 (IQR 15.0–30.0) to 8.0 (IQR 5.0–15.0; RR: ≥50% 55.5%; ≥75% 22.4% and 100% 1.1%.	Constipation (8.8%), usually rated as mild; severe in one case and classified as a SAE.
Scheffler et al., 2020 [24]	EM/CM: 26/74	12 weeks	R S N = Germany	RR ≥50%	% of conversion CM → EM; improvement of intensity and duration of pain; % AEs.	*Primary endpoints:* EM: 57.7%; CM: 41.9%. *Secondary endpoints:* 53% CM → EM; 70.5% and 58.9% improvement of intensity and duration of pain, respectively.	AEs: 42%: 23.8% constipation; 23.8% injection side skin symptoms or itching; 16.7% fatigue or a feeling of exhaustion; 9.5% insomnia.
Ornello et al., 2020 [25]	CM: 91	24 weeks	P M (n = 7) N = Italy	% of conversion to EM from baseline to months 4–6 of treatment and during each month of treatment.	Change in MHD, AMD, and NRS.	*Primary endpoints:* 12.1% discontinuation before month 6 due to ineffectiveness, 68.1% CM → EM. *Secondary endpoints*: MHD from 26.5 (IQR 20–30) to 7.5 (IQR 5–16; *p* < 0.001), AMD from 21 (IQR 16–30) to 6 (IQR 3–10; *p* < 0.001), and NRS from 8 (IQR 7–9) to 6 (IQR 4–7; *p* < 0.001). Significant decreases both in converters and in non-converters.	1 pt discontinued the treatment before month 6 for AE.
Russo et al., 2020 [26]	CM: 90 (failure to ≥4 migraine preventive medication classes)	24 weeks	P S N = Italy	≥30% reduction in MHD, after ≥3 months of therapy switched to monthly erenumab 140 mg.	Disease severity, migraine-related disability, and impact and validated questionnaires to explore depression/anxiety, sleep, and QoL. Pain Catastrophizing Scale, Allodynia Symptom Checklist-12, and MIGraine attacks-Subjective COGnitive impairments scale (MIG-SCOG).	*Primary endpoints:* After 3 doses of 70 mg 70% R, 30% switched to 140 mg; after 6 doses 29% R. After 3 doses, MHD −9.7 (*p* < 0.001) and after 6 doses, −12.2 (*p* < 0.001). RR: ≥50% of MHD after 3 and 6 doses: 53% and 70%; *Secondary endpoints*: Pain severity, migraine-related disability, and impact on daily living, QoL, Pain Catastrophizing Scale, Allodynia (all *p* < 0.001) Scales, quality of sleep, and symptoms of depression or anxiety (*p* < 0.05), but not MIG-SCOG, also improved.	No new AE was reported.
Lambru et al., 2020 [27]	CM: 162	24 weeks	P S N = UK	Change in MMD at weeks 24 vs. baseline.	RR: 30%, 50%, 75%; % stopped MO HIT-6 score.	*Primary endpoints:* MMD: −7.5 (*p* < 0.001); MHD: −6.8 (*p* < 0.001); *Secondary endpoints*: RR: 60%, 38%, 22%; MO: 54% → 25%; HIT-6: −7.5 (*p* = 0.01).	At least one AE reported by 48% at month 1, 22% at month 3, and 15% at month 6. The most frequent AEs: constipation (20%) and cold/flu-like symptoms (15%).
Barbanti et al., 2021 [4]	HFEM/CM: 60/182	48 weeks	P M (n = 15) N = Italy	Change in MMD and MHD at weeks 45–48 vs. baseline.	Change in MAI ≥50%, ≥75%, 100%, RR, and any variation in VAS and HIT-6 scores at weeks 45–48.	221 considered for effectiveness, 242 for safety. *Primary endpoints:* HFEM: MMD −4.3; CM: MHD −12.8 *Secondary endpoints*: HFEM: VAS −1.8; HIT-6 −12.3; MAI from 11.0 ([IQR] 10.0–13.0) to 5 (IQR 2.0–8.0); RR: ≥50% 56.1%; ≥75% 31.6%; 100% 8.8%; CM: VAS −3.0; HIT-6–13.1; MAI from 20.0 (IQR 15.0–30.0) to 6.0 (IQR 3.8–10.0) RR: ≥50% 75.6; ≥75% 44.5%; 100% 1.2%.83.6% CM → EM.	AEs: 18.6% usually mild. The most common: constipation 10.3%, injection site erythema 3.3%. 1.2% of patients experienced SAEs: 1) Paralytic ileus (treatment-related) 2) Non-ST segment elevation myocardial infarction (not related) 3) Myocardial infarction (not related).
Ornello et al., 2021 [28]	HFEM/CM: 374/1036	12 weeks	R M (n = 16) I = Italy, UK, de, Czech Republic, Russian Federation, Australia.	RR: 0–29%, 30–49%, 50–75%, and ≥75%. Comparison between men and women.	NA	*Primary endpoints:* RR ≥ 75%: 20.2%; RR:50–74%: 20.7%; RR:30–49% 15.3%; RR:0–29%: 31.4; *Secondary endpoints*: Gender did not influence the efficacy of outcomes.	NA
de Vries Lentsch et al., 2021 [29]	HFEM/CM: 54/46	24 weeks	P M (n = 2) N = Netherlands	MMD after 6 months vs. baseline.	AMD, RR, well-being, and coping with pain.	*Primary endpoints:* MMD: −4.8 (*p* <0.001); *Secondary endpoints*: AMDs (*p* <0.001) in all months; RR ≥50%: 36% in ≥3/6 months, and 6% in all 6 months; RR ≥30%, 60%, and 24%, respectively. Well-being (*p* < 0.001) and coping with pain (*p* < 0.001).	AEs: 93%. Most common: abdominal complaints 72%, including constipation 65%, fatigue 43%, and injection site reactions (27%).
De Matteis et al., 2021 [30]	HFEM+CM:32	52 weeks 8-weeks follow-up after treatment completion	P M (n = 2) N = Italy	RR and change in MMD during weeks 1–4 after treatment completion as vs. baseline and the last 4 weeks of treatment.	RR and changes in MMD, AMD, and NRS in who did not restart treatment during weeks 5–8 after treatment completion vs. last 4 weeks of treatment and with baseline.	*Primary endpoints:* RR ≥50%: 56%; RR 50–75%: 34%; RR 75–100%: 22%; MMD: −19 (*p* < 0.001) last 4 weeks of treatments, −15 (*p* < 0.001) weeks 1–4 after treatment completation. *Secondary endpoints*: AMD, NRS: during the last 4 weeks of treatment (*p* < 0.001); weeks 1–4 after completion (*p* < 0.001) lower than baseline (MMD and AMDs *p* < 0.001, NRS *p* = 0.005). 56% RR ≥ 50% from baseline. At week 4 after treatment completion, 31% restarted treatment due to disease rebound.	NA
Faust et al., 2021 [31]	CM: 1043	12 weeks	R M N = US	Change in MHD/MMD, MIDAS, and HIT-6 after 3 months vs. baseline.	NA	MMD/MHD: −5.64 MIDAS: −6.89 HIT-6: −3.87	Constipation: 2% (n = 23 pts).
Andreou et al., 2022 [32]	CM: 135	2 years	P S N = England	Sustained effectiveness in 24 months of treatment.	MMD, HIT-6 at months 6, 12, and 18.	*Primary endpoints:* RR: 30%: 23%; RR: 50% and 75%, 16%, and 8%, respectively. *Secondary endpoints*: MMD: (*p* < 0.001) HIT-6: (*p* < 0.001) at all timepoints.	NA
Becker et al., 2022 [33]	HFEM/CM: 31/64	24 weeks	P S N = Canadia	≥50% reduction in MMD at week 12 from baseline.	Effectiveness at week 24: change in MMD; patient-reported outcomes (PROs); Clinical Global Impressions-Severity scale (CGI-S); and the Clinical Global Impressions-Improvement scale (CGI-I) at weeks 12 and 24, respectively.	*Primary endpoints:* RR: 50%, 33.7% *Secondary endpoints*: MMD: −4.9 at weeks 12 and −5.7 at weeks 24 PROs: responses increased at follow-up timepoints CGI: 65.3% and 52.3% improvement at weeks 12 and 24, respectively.	AEs: 24% SAEs: 1% Discontinution: 3.1%.
Pensato et al., 2022 [34]	CM+MOH: 149 (previously failed onabotulinum toxin A)	12 weeks	P M (n = 5) N = Italy	RR 50%, 75%.	MHD, MAI, and CM →EM	*Primary endpoints:* RR ≥50%: 51%; RR ≥75%: 20%. *Secondary endpoints*: MHD: −11.3 (*p* < 0.001) MAI: −29.3 (*p* < 0.001) CM → EM: 64%	No SAEs observed.
Cullum et al., 2022 [35]	CM: 300	52 weeks	P S N = Denmark	≥30% reductions in MMDs from baseline to week 9–12.	≥50% reductions in MMDs from baseline to weeks 9–12.	*Primary endpoints:* RR ≥30%: 71.4% *Secondary endpoints*: RR ≥50%: 6.4%	AEs: 73.3% Constipation: 41.3% Injection site reaction: 9.7% Nausea: 7.3% Fatigue: 6.7% Aggravation of migraine: 4.7% Tinnitus: 4.7% Alopecia: 3.7% Muscle cramps: 3.7% Dizziness: 3.3% Abdominal pain: 3% Insomnia: 2.7% Metrorrhagia: 2% Weight gain: 2% Hot flushes: 2% Flushing: 2% Severe AEs: one pts (0.3%) pulmonary embolism—no discontinution; 7.3% discontinuation fof costiation.
Khalil et al., 2022 [36]	CM: 92	12 weeks	P S N = UK	Change in MMD at 3 and 6 months; RR 30% and 50% at 3 months.	Change in MHD, MAI, and HIT-6 at 3 and 6 months.	*Primary endpoints:* MMD: −4 and −9 at 3 and 6 months, respectively (*p* < 0.001) RR 30% and 50%; 53% and 36% at 3 months, respectively. *Secondary endpoints*: MHD: −7 and −5 (*p* < 0.001) MAI: −1 and −5 (*p* < 0.001) HIT-6: –3 and −5 (*p* < 0.001) at 3 and 6 months, respectively.	AEs 41% Constipation: 50% Worsened headache: 19% Skin reaction:14% Dizziness:14% Cramps: 10% Bloating: 7% Nausea: 7% Fatigue: 7% No SAEs reported 8% stopped for lack of efficacy.
Alsaadi et al., 2022 [37]	EM/CM 95/71	24 weeks	R M (n = unspecified) N = United Arab Emirates	Change in MMD/MHD at month 1, 3, and 6.	RR: ≥50%, ≥75%, 100%. Change in AMD, % MO at months 1, 3, and 6.	*Primary endpoint:* MHD:−8.4; −11.1 and −11.6 at months 1, 3, and 6 respectively; *Secondary endpoints*: RR: ≥50% ranging between 80 and 91% at all timepoints. RR ≥ 75%: 39.1%, 46.5%, and 52.6% at months 1, 3, and 6, respectively; RR 100%: 5.7%, 14.1%, and 9.5% at the same timepoints. AMD −8.7; −9.9; −11 at months 1, 3, and 6, respectively. MO: −17.5%; −23.5%, −100%.	AEs 20.4%: constipation 3%, insomnia 2.4%, influenza 1.8%, followed by falls, dizziness, arthralgia, stress, and headaches (1.2% each). 76.5% of AEs were mild to moderate in severity. Two patients (1.2%) had severe AEs: atypical pneumonia and spontaneous abortion in one (0.6%) patient each.
Ornello et al., 2022 [38]	HFEM+CM: 1215	9–12 weeks	P M (n = 16) I = Italy, UK, Germany, Czech Republic, Russian Federation, Australia.	RR: 0–29%, 30–49%, 50–74%, and ≥75% at weeks 9–12 vs. baseline. For each response category, median MMD and HIT-6 at baseline and at weeks 9–12.	Categorization of residual MMD at weeks 9–12: 0–3, 4–7, 8–14, ≥15. Four categories of HIT-6: ≤49, 50–55, 56–59, and ≥60. Calculations in men and women.	*Primary endpoints:* RR 0–29%: 31.4%; RR 30–49%:15.3%; RR 50–74%: 32.6%; and RR ≥75%: 20.7%. *Secondary endpoints*: 0–3 residual MMD: 20.2%, 4–7: 36.5%, 8–14: 24.6%, ≥15: 18.7%. of R (4–7 MMD) 50–74%: 62.1% and (8–14) 23.7%; of R (0–3) ≥75%: 74.2% (4–7) 25.8%. No differences in gender for residual MMD; HIT-6 distribution is less favorable in women in the 0–29% (*p* = 0.004) and in the 30–49% (*p* = 0.003) response categories.	NA
Gantenbein et al., 2022 [39]	EM+CM: 172	24 weeks	P M (n = 13) N = Switzerland	Impact on QoL, migraine-related impairment, and treatment satisfaction HIT-6, mMIDAS, Impact of Migraine on Partners and Adolescent Children (IMPAC), TSQM-9 (Treatment Satisfaction Questionnaire for Medication) after 6 months.	NA	HIT-6 −7.7 (*p* < 0.001), the mMIDAS −14.1 (*p* < 0.001), MMD −7.6 (*p* < 0.001), and AMD −6.6 (*p* < 0.001). IMPAC: −6.1 (*p* < 0.001). Mean effectiveness of 67.1, convenience of 82.4, and global satisfaction of 72.4 of patients in the TSQM-9.	99 AEs and 12 SAEs were observed in 62 and 11 patients, respectively. All SAEs are not related to the study medication.
Cetta et al., 2022 [40]	15 over 65 (O65) and 15 under 65 (U65), matched for sex HFEM/CM:12/18	24 weeks	P S N = Italy	Change in MHD and MMD vs. baseline between young and elder migraine patients.	MAI, AMDs, HIT-6, MIDAS, NRS, and ASC-12 after 3 (M3) and 6 (M6) months of treatment.	*Primary endpoints:* baseline MHD and MMD of both groups: 20. Mean age was 70 (65–76) and 45 (19–55) in the O65 and U65 groups, respectively. At M3 and M6, there are no statistical differences between groups. *Secondary endpoints*: at M3 and M6, reduction of all clinical features under examination, without statistically significant differences between the two groups.	Similar proportion of AEs (M3 and M6, *p* = 1.0) in each group.
Lanteri-Minet et al., 2023 [41]	CM 128	52 weeks	P M (n: unknown) N = France compassionate erenumab use program	RR≥ 50% at weeks M3, M6, M9, and M12.	Change in MMD, MHD, HIT-6, RR ≥ 75%, RR ≥ 30%, CM → EM, MO → non-MO, PGIC at M3, M6, M9, and M12.	*Primary endpoint:* RR ≥ 50% at M3, M6, and M9. and M12: 52.9%, 58.5%, 57.%, and 58.8%, respectively. *Secondary endpoints:* MMDs: the median (IQR) was 18.0 (13.0–26.0), 9.0 (5.0–17.0), 7.5 (5.0–14.0), 8.0 (5.0–12.5), and 8.0 (5.0– 12.0) at M0, M3, M6, M9, and M12, respectively MHDs: the median (IQR) was 23.0 (16.0–30.0), 11.0 (6.0–22.3), 11.0 (6.0–19.0), 9.0 (6.0–16.0), and 9.0 (6.0– 15.8); HIT-6 score, the median (IQR) was 68.0 (63.8–73.3), 60.0 (54.0–65.0), 60.0 (50.3–53.0), 59.0 (50.0–63.0), and 58.0 (50.0–62.9); RR ≥ 75%: 15.7%, 17.8%, 23.4%, and 23.5%; RR ≥ 30%: 81.4%, 80.5%, 79.4%, and 83.3%; CM: 37.9%, 34.7%, 29.9%, and 24.5% compared to baseline (88.6%). MO 48.6%, 43.2%, 40.2%, and 38.2% compared to baseline (90.7%).	AEs: 37.9% Cutaneous erythema and/or pain at the injection site (30%); constipation (15.7%); muscle spasm (1.4%); alopecia (0.7%); and blood pressure increase (0.7%). No SAE.
Troy et al., 2023 [42]	CM: 177	17–30 months	P M (n = 4) I = Ireland, UK, USA	PROM/QoL outcomes over a period of 17–30 months.	HIT-6, MIDAS, and MSQ before starting treatment and at intervals of 3–12 months after starting treatment.	*Primary endpoints:* 61.6% significant improvement after 6–12 months. 54.8% on treatment (median of 25 months). *Secondary endpoints:* From baseline to 25–30 months: HIT-6: −14; MIDAS: −101; MSQ: −30.	38.4% stopped during the first year, due to lack of efficacy and/or possible AEs.
Pilati et al., 2023 [43]	CM: 88 (CM+MO: 84)	12 weeks	P M (n = 6) N = Italy	Variation in MEQ, PSQI, SCI, (Sleep Condition Indicator), ESS, MIDAS, and HIT-6 at T3 and later vs. baseline.	Changes in MMD, DSMs, RR 30%, 50%, 75%, and 100% after the first dose;	*Primary endpoints:* MEQ morningness → intermediate: *p* < 0.05; PSQI score > 5 at baseline in 64% of patients and no variation at follow-up. SCI significant increase at T3 (*p* = 0.0144) not confirmed during later (*p* < 0.05). ESS no statistical significance during follow-ups. At T3 MMD: −10.6 (*p* < 0.001) in patients receiving 70 mg and −16.4 (*p* < 0.001) in 140 mg (*p* < 0.001). A significant difference between T3 and T9 (*p* = 0.014) was not confirmed in T3 vs. T12 (*p* = 0.766). *Secondary endpoints:* After the first dose of 70 and 140 mg (T1), RR ≥30%: 13% and 18%; RR ≥50%: 29% and 34%; RR≥ 75%: 13% and 26%; and RR 100%: 0% and 3%, respectively. MIDAS and HIT-6 during all the evaluations vs. baseline (*p* < 0.05).	10 different AEs in 37.5%. The most common: constipation in 10.2%. No AE led to withdrawal. 5.7% complained of insomnia.
Buture et al., 2023 [44]	82 New Daily Persistent Headache and Persistent Post-Traumatic Headache	over a two to three years	R M (n = 3) I= Ireland, UK, USA	Improvement of QoL after 30 months vs. baseline.	NA	*Primary endpoints:* significant improvements in QoL in 1/3 over a period of 11–30 months, with a 35% persistence after a median of 26 months of treatment.	NA

**Table 2:** Summary of changes in MMD, MHD, NRS, MAI, MIDAS, and HIT-6 scores, as well as ≥50%, ≥75%, and 100% response rates. Table 3 reports information on adverse events.

	3 Months			6 Months			12 Months		
	All	EM	CM	All	EM	CM	All	EM	CM
MMD	-	−2.5/−4.5	-	-	−4.8/−7.7	-	-	−4.3/−7.8	-
	-	−4.4	-	-	−7.5 −8.2	-	-	−6/−11.5	-
	-	−4.6/−7.3	-	-	−6.9, −7.7	-	-	−6.4/−6.5	-
MHD	−6.9/−7.9	-	−4.7/−15	−4	-	−6.8/−19	-	-	−12.8/−21.7
	-	-	−7.3/−15	−10.2/−14.2	-	−11/−14.9	-	-	−10/−11.9
	-	-	−8.2/−9.4		-	−9.7/−14.2	-	-	−14.5/−15
NRS	-	−0.5/−1.9	−1.7/−3	-	−0.7/−2	−2/−3	-	−0.7/−3	−1.8/−3.6
	−1.6/−2.1	−1/−2	−1	−1.4/−2–7	−2	−2	-	−2	−1.9/−2.8
	-	−3.1	−2.5	-	−3.4	−2.7	-	−3.4	−3.4
MAI	−6.5/−7.6	−5/−7	−12/−15	−1.7	−5/−8	−14/−15	-	−5/−8	−14/−16
	−4.1	−4/−6.5	−4/−15	−14.2/−17.5	−8	−8/−29.7	-	−9	−18.4/−30.1
		−5.7	−11.1	-	−8	−15.1		−6/−7	−15.5/−16.5
MIDAS	-	−28.5/−48.9	−35.1/−42.1	-	−32.4/−44.6	−37.1/−45.9	-	−38.3/−47	−44.3/−65.1
	−32.9	-	−14/−71.8	−64/−77.5	−27	−54	-	−9.3	19.5/−57.6
	-	−31/−53.8	−43.7/−55	-	−55	−72.6	-	−18/−50.4	−53.5/−76.6
HIT-6	−7/−8.4	−8.4/−10.7	−9.7/−11.4	-	−7.1/−13.3	−7.5/−12.7	-	−12.3/−13.7	−13.1/−14
	−4.4	−4	−11	−9.3/−14.6	−63	−50	-	−12.3	−13.7/−58.4
	-	−10/−18.1	−0.3/−28	-	−20.9	−24.3	-	−16.9/−18.5	−15/−17.9
≥50% RR (%)	41.9/53.3	57.7/59.4	41.9/55.5	-	36/63	22/70	-	56/85	44.5/68
	50.9	41.7/76	48.1/76.5	73.2/95.4	76.7	61.5/74.4	-	73.8	60.5
		43.3/76.5	38.3/58.3	-	75/90.4	52.2/76.3	-	75.5/78.6	71.6/75.9
≥75% RR (%)	20.2/20.7	16.8/22.9	20/22.4	-	16.3/38.4	38/42.3	-	31.6/42	31.6/44.5
	27.3	41.7/73.8	27.7/44.2	45.7/55.8	30.2	63.5	-	37.2	38.1
		24/40.2	17/25	-	30.8/36.5	14.9/44.8		35.7/36.7	44.4/37
100% RR (%)	-	1/3	1.1/5	-	4.6	2.8–9	-	8.8	1.2/ 8.5
	-	7/20	3.4/10.8	-	9.3/11.6	4.7	-	2.3	3.4
	-	9.9	0	-	9.6	1/1.5	-	2/14.3	3.7/5.6

All: migraine patients (not specified if episodic or chronic); EM: episodic migraine; CM: chronic migraine; MMD: monthly migraine days; MHD: monthly headache days; NRS: numerical rating scale score; MAI: monthly analgesic intake; MIDAS: migraine disability assessment scale score; HIT-6: Headache Impact Test-6 score; ≥50% RR: ≥50% response rate; ≥75% RR: ≥75% response rate; 100% RR: 100% response rate.

**Table 3:** Overview of adverse events reported in multiple mAbs REW studies.

		AE Type	Frequency Range
Pts with AEs	0–54.5%	- injection site reaction	0–30%
		- constipation	0–54.5%
		- dizziness	0–3.3%
		- fatigue	0–4%
Pts with SAEs	0%		
Pts who discontinued treatment due to AEs	0%		

RWE studies have consistently reported overall good safety and tolerability profiles for erenumab. The proportion of patients reporting adverse events (AEs) ranged from 7.8% to 93% (median 23.5%). The most frequently reported AEs were constipation (median 15.2%), fatigue (median 16.7%), and injection site erythema (median 13.5%) (Table 4). Five serious AEs were reported, accounting for 0.08% of cases. Among these, three were treatment-related: paralytic ileus in one patient and severe constipation in two patients, totaling 0.05%. The other two cases, myocardial infarction, were not treatment-related. The discontinuation rate ranged from 0% to 40% (Table 4).

(b) Fremanezumab

We identified eight relevant articles focusing on fremanezumab in RWE studies, involving 1776 patients (701 with EM and 1075 with CM; 397 males and 1379 females; medication overuse: 579), with a mean age ranging from 38.5 to 49.5 years (Table 5) [6,45,46,47,48,49,50,51]. Among these studies, seven were prospective and one was retrospective (Figure 3). Of the prospective studies, two were conducted at single centers [49,50], while five were multi-center studies. The retrospective studies were multi-center [46]. The durations of the studies varied, with three lasting 3 months [45,48,49], three lasting 6 months [46,47,50], and two extending to 12 months [6,51].

Participants’ disease duration ranged from 7 to 29.6 years; baseline MMD/MHD spanned from 10 to 24.3; NRS from 7.9 to 9.6, MAI from 10 to 12 in EM; and 20 to 22.5 in CM. HIT-6 score ranged from 62.4 to 68 in EM and 68.3 to 70 in CM, while MIDAS score ranged from 66 to 73.1 in EM and 89.4 to 98 in CM (Table 5). Prior treatment failures among participants ranged from 4.3 to 6. Variations in MMD, MHD, NRS, MAI, MIDAS, and HIT-6 scores, ≥50%, ≥75%, and 100% response rates and AEs are outlined in Table 2. A proportion of 61.2% to 93.8% of individuals transitioned from CM to EM, while 75% to 96.6% of patients discontinued medication overuse.

AEs, categorized as mild and transient, were observed in a range from 2.4% to 23% of the individuals (with a median of 9.6%) (Table 6). The predominant occurrences included reactions at the injection site (median 4.7%) and constipation (median 2%). The proportion of patients discontinuing the treatment spanned from 2% to 31% (median 9.5%). The reasons for discontinuation included lack of effectiveness (14%), personal reasons (6%), lost to follow-up (6%), AE (2%), pregnancy (1%), lack of compliance (1%), and other unspecified reasons (31%) (Table 6).

(c) Galcanezumab

We identified 15 relevant articles focused on evaluating Galcanezumab in RWE settings (Table 7) [7,52,53,54,55,56,57,58,59,60,61,62,63,64,65]. 

The total number of individuals involved was 2150, comprising 248 with EM and 1902 with CM. The gender distribution was 844 males and 1306 females, with a mean age of 44.9 years. The number of patients with calculated medication overuse was 822. Among the 15 studies, 11 were prospective [7,53,54,55,56,57,58,59,60,62,64] and 4 were retrospective (Figure 3) [52,61,63,65]. All retrospective studies were single-center, while among the prospective studies, seven were single-center [56,57,58,59,60,62,64] and four were multicenter [7,53,54]. The duration of the studies varied, with seven lasting 3 months [52,54,58,59,61,62,65], five lasting 6 months [53,56,57,63,64], and three lasting 12 months or more [7,55,60].

Participants exhibited diverse disease durations, spanning from 24 to 30.1 years, with baseline MMD/MHD ranging from 11.2 to 21. The MAI spanned from 12 to 24.7, NRS from 7 to 7.6, MIDAS score from 30 to 96.1, and HIT-6 score from 64.6 to 66.9. Prior treatment failures ranged from 3 to 12 among participants (Table 7). Variations in MMD, MHD, NRS, MAI, MIDAS, and HIT-6 scores, ≥50%, ≥75%, and 100% response rates, along with adverse event details, are documented in Table 2. A proportion of 52.3% to 77.2% [26,27,28,29,30,31,32,33,34,35,36,37,38,39,40,41,42,43,44,45,46,47,48,49,50,51,52,53,54,55] of individuals transitioned from CM to EM, while 61.8% to 82.0% of patients discontinued medication overuse [54]. 

AEs, rated as mild and transient, occurred in 0% to 26.9% of the patients (median 2.3%). The most common were injection site reactions (median 8%) and constipation (median 7.7%) (Table 8). No serious AEs were reported, nor did any patient discontinue treatment due to adverse events. The proportion of patients discontinuing the treatment ranged from 5.4% to 22.5% (median value: 9.8%). The reason for discontinuation was detailed in only three studies, and in all cases, it was due to a lack of efficacy, ranging from 6.1% to 14.2%. Among patients who discontinued therapy for reasons other than adverse events, no dropouts were reported in the 12-week studies. In the 24-week studies, nine dropouts were reported in one study and 3 in another [54,60]. In a 1-year study, one patient discontinued treatment due to drug unavailability [7].

(d) *RWE studies examining multiple monoclonal antibodies*

Fourteen RWE studies explored more than one anti-CGRP mAb [8,9,10,11,12,13,14,15,16,17,18,19,20,21]. Among these, ten studies encompassed erenumab, fremanezumab, and galcanezumab (Table 9), [10,11,13,14,15,21].Two studies focused on both erenumab and galcanezumab [8,9], another on erenumab and fremanezumab [12], and one on galcanezumab and fremanezuma [16]. Of these studies, eight were prospective [8,9,10,11,14,18,19,20,21] and five were retrospective [12,13,15,16,17]; nine were conducted at a single center [8,10,11,12,13,15,16,17,19], and four were multicenter [9,14,15,17,18]. The combined patient cohort across these RWE studies, investigating more than one anti-CGRP mAb, totaled 6231 globally (females 5270, males 961; EM: 1786; CM: 4463; mean age: 46.4).

## 4. Discussion

Our review encompasses 61 studies involving 18,097 migraine patients, evaluating the real-world effectiveness and tolerability of anti-CGRP mAbs across various populations in Europe, America, and Asia. Most of these studies were single-center and prospective. Erenumab emerged as the most extensively investigated anti-CGRP mAb, being the first to enter the market.

This enthusiasm is driven by the introduction of the first specific and selective drugs for migraine prevention, which demonstrate a notable efficacy-to-tolerability ratio. However, we observed considerable heterogeneity among the studies regarding sample size, inclusion criteria, socio-demographic details, migraine clinical features, and endpoint definitions, which is typical of RWE investigations.

In general, all the studies demonstrated the remarkable effectiveness of anti-CGRP mAbs, coupled with very good tolerability and safety. Patients experienced significant reductions in migraine frequency and severity, with minimal adverse effects reported.

*Effectiveness*. Nearly all studies assessed effectiveness as the primary endpoint [5,6,7,8,9,10,11,12,13,14,15,16,17,18,19,20,21,22,23,24,25,26,27,28,29,30,31,32,33,34,35,36,37,38,39,40,41,42,43,44,45,46,47,48,49,50,51,52,53,54,55,56,57]. They reported significant MMD, MHD, MAI, NRS, HIT-6, and MIDAS scores. For instance, reductions in MMD ranged from 2.5 to 7.3 at week 12, from 4.8 to 8.2 at week 24, and from 4.3 to 11.5 at week 48. Similarly, changes in MHD ranged from −4.7 to −15 at week 12, from −4 to −19 at week 24, and from −10 to −21.7 at week 48 (see Table 2). Comparatively, these reductions are more pronounced than those observed in 12-week RCTs with erenumab (−1.8), fremanezumab (−3.7/−4.1), and galcanezumab (−4.1). These improvements were associated with a substantial proportion of responders and super-responders. The proportion of patients achieving ≥50% response rates ranged from 41% to 76.5% at week 12, from 36% to 90.4% at week 24, and from 44.5% to 85% at week 48. For ≥75% responders, the corresponding figures were from 20.2% to 73.8% at week 12, from 14.9% to 63.5% at week 24, and from 31.6% to 44.5% at week 48. The percentage of patients achieving a 100% response ranged from 0% to 20% at week 12, from 1% to 11.6% at week 24, and from 2% to 14.3% at week 48.

The higher responder rates in RWE studies compared to RCTs might be attributed to a higher placebo effect in real-world settings, where patients have a more familiar relationship with their healthcare providers [3]. Additionally, real-world patients might have increased CGRP activity, potentially enhancing the effects of anti-CGRP treatments. Indeed, migraine patients in daily clinical practice often exhibit higher migraine frequency and more frequent depressive comorbidities, which are conditions putatively associated with elevated CGRP levels [53,54].

*Tolerability and safety*. Overall, anti-CGRP mAbs were well tolerated. Fremanezumab and galcanezumab demonstrated a lower incidence of adverse events compared to erenumab, particularly regarding constipation and fatigue [4,24,27,29]. Serious adverse events were exceedingly rare (0.05%) and were limited to patients receiving erenumab, including cases of ileus paralyticus (1 patient), severe constipation (2 patients), and myocardial infarction (2 patients) [4]. The lower prevalence of adverse events in RWE compared to RCTs could be due to less rigorous surveillance in routine clinical practice and the possibility that patients with a history of failing multiple traditional treatments—frequently associated with debilitating adverse events—might underreport mild or transient adverse events.

Notably, real-life investigations have also disclosed several insightful clinical aspects that have not been addressed by RCTs, such as reduced pain severity, predictors of response, late response, and effects of treatment discontinuation on migraine frequency.

*Reduction in pain intensity*. Real-life studies focusing also on pain intensity reported reductions in NRS scores of up to −3.1 at 12 weeks [47], −3.4 at 24 weeks [49], and −3.6 at 48 weeks [4]. This approximately 30% reduction in pain severity signifies a meaningful clinical improvement, correlating with reduced disability and interictal migraine burden. These findings highlight the need for dedicated clinical trials to explore these outcomes further.

*Predictors of treatment response*. Symptoms of trigeminal peripheral sensitization, such as unilateral pain and cranial unilateral autonomic symptoms, either alone or in combination with ictal allodynia, were identified as potential positive predictors of response in real-life studies. Conversely, overweight and obesity were found to be negative predictors of response [5].

*Timing for treatment response.* Despite their typically rapid onset of action, late responses (>12 weeks) to anti-CGRP mAb treatment have also been documented [14]. More than 50% of patients who initially exhibited a <50% response rate at week 12 had become ≥50% responders by week 24. Late responders tend to have a higer BMI, more frequent prior treatment failures, psychiatric comorbidities, and less frequent experience of unilateral pain alone or with unilateral cranial autonomic symptoms or allodynia. The presence of the phenomenon of late response suggests reconsidering the time interval for evaluating the response to anti-CGEP mAbs and, in general, rethinking the criteria for defining resistance/refractoriness to preventive treatments [66].

*Rebound of migraine frequency after treatment discontinuation*. Following a 12-month treatment cycle with anti-CGRP mAbs, a significant increase in migraine frequency within 4 weeks after discontinuation compared to the last month of treatment was documented, although it did not return to baseline levels [9]. This finding suggests that a short-term treatment with anti-CGRP mAbs does not modify the course of migraine.

A limitation of RWE studies is the potential for selection bias due to a reduction in subjects with longer follow-up periods. This may have skewed the results toward favorable outcomes, as individuals who did not benefit or who experienced adverse effects likely discontinued the study earlier, potentially leading to an overestimation of the long-term efficacy and tolerability of mAbs treatments for migraine.

## 5. Conclusions

This comprehensive narrative review of RWE studies highlights the significant interest among clinicians and researchers in anti-CGRP mAbs as pioneering specific and selective drugs for migraine prevention. It underscores the importance of real-life studies, not only for assessing their efficacy and tolerability but also for identifying clinical aspects that RCTs may not capture—such as reduction in pain severity, predictors of response, and late response—especially in a multifaceted and complex migraine population.

Despite the variability in study designs and patient characteristics, the overall results suggest that the effectiveness and tolerability of anti-CGRP mAbs in real-world applications may exceed those observed in RCTs, an extraordinary finding in clinical neurology. These data should inspire further investigations to optimize the clinical use of anti-CGRP mAbs.

## Figures and Tables

**Table 4:** Overview of adverse events among patients receiving erenumab in RWE studies.

		AE Type	Frequency Range	Frequency < 8% (pts n)	Frequency 8–10% (pts n)	Frequency > 10% (pts n)
Pts with AEs	0.6–93%	- constipation	8.8–65%	23	1	4
		- injection site erythema	0.8–27%	1	-	2
		- fatigue	0.8–43%	1	-	2
		- insomnia	5.7–9.5%	-	1	-
		- cold/flu-like symptoms	5–15%	-	-	1
		- dizziness	0.6–1.8	1	-	-
		-arthralgia	1.1–1.7	2	-	-
Pts with SAEs	0–1.2%	- paralytic ileus - constipation - myocardial infarction	0.4% 0.8% 0.8%			
Pts who discontinued treatment due to AEs	0–1.2%	- paralytic ileus - constipation - myocardial infarction	0.4% 0.8% 0.8%			

**Table 5:** Fremanezumab in RWE studies.

Author/ Year	N° of pts	Observation Period	Study Type/Center/ National–International	Primary Endpoint	Secondary Endpoint	Results	Safety Findings
Barbanti et al., 2022 [45]	67 (HFEM/CM: 21/46)	12 weeks	P M (n = 9) N = Italy	Change in MMD for HFEM and MHD for CM at weeks 9–12 vs. baseline.	Change in MAI, NRS, HIT-6 and MIDAS and ≥ 50%, ≥ 75% and 100% RR at the same time intervals.	*Primary endpoints:* MMD: −4.6 (*p* < 0.05) MHD: −9.4 (*p* < 0.001). *Secondary endpoints:* MAI: −5.7 (*p* < 0.05), −11.1 (*p* < 0.001); NRS: −3.1, −2.5 (*p* < 0.001) MIDAS: −58.3 (*p* < 0.05), −43.7 (*p* < 0.001) in HFEM and CM, respectively; HIT-6: −18.1 (*p* < 0.001) in HFEM. The ≥ 50%, ≥ 75%, and 100% RR at week 12 were 76.5%, 29.4%, and 9.9% in HFEM and 58.3%, 25%, and 0% in CM	5.7% reported TEAEs: 1 injection site erythema (1.9%), 1 abdominal pain (1.9%), and 1 neck pain and somnolence (1.9%).
Driessen et al., 2022 [46]	1003 (HFEM/CM: 416/587)	24 weeks	R M (n = 421 clinicians: 240 neurologists, 80 general practitioners, 36 pain management specialists, 21 psychiatrists, 38 PAs or NPs, and 6 other headache specialists) N = Netherlands	Changes in MMD and MHDs at month 6	NA	MMD/MHD: −7.7 and −10.1 in HFEM and CM, respectively; −10.8 in the MO sub-group; −9.9 in the MDD subgroup, −9.5 in the GAD subgroup, and −9.0 in the prior exposure to a different CGRP mAb subgroup.	NA
Barbanti et al., 2023 [47]	410 (HFEM/CM: 214/196)	24 weeks	P M (n = 28) N = Italy	Change in MMD and MHD at weeks 21–24 vs. baseline	Changes in MAI, NRS, HIT-6, MIDAS, and ≥50%, ≥75%, and 100% RR at weeks 21–24 vs. baseline.	*Primary endpoints:* MMD: −6.9 (*p* < 0.001) MHD: −14.2 (*p* < 0.001) *Secondary endpoints*: ≥50%, ≥75%, and 100% responders: HFEM: 75.0, 30.8, and 9.6%; CM: 72.9, 44.8, and 1%; NRS: −3.4, −2.7; MAI: −8.0, −15.1; HIT: −6, −8.0; −20.9, −24.3; MIDAS: −55.0, −72.6, respectively, in HFEM and CM.	NA
Argyriou et al., 2023 [48]	204 (HFEM/CM:107/97)	12 weeks	P M (n = 6) N = Greece	A minimum 50% decrease in MHD at T1 vs. T0 and the percentage of 30%, 75%, and 100% reduction in mean MHD	Changes in mean MHD, migraine severity, mean days with intake of any acute headache medications, MIDAS, HIT-6, EQ-5D, and QOL	*Primary endpoints:* reduction MHD: 83.5% HFEM and 62.6% CM patients. *Secondary endpoints:* MHD: −6.5, −9.4, respectively, in HFEM and CM; *p* < 0.001 in migraine severity, mean days with intake of any acute headache medications, MIDAS, HIT-6, EQ-5D, and QOL	25% of patients (n = 26) experimented treatment-associated toxicity: 43.8% (n = 21) erenumab versus 16.3% (n = 7) galcanezumab versus 15.4% (n = 2) fremanezumab.
Cullum et al., 2023 [49]	91 (HFEM/CM:-/91)	12 weeks	P S N = Denmark	Reduction ≥30% in MMD from baseline to weeks 9–12.	Responders ≥50 and ≥75% and proportion of patients reporting AEs.	*Primary endpoints:* MMD: −7.3; MHD: −8.2. ≥30% RR: 65% *Secondary endpoints:* ≥50 and ≥75% RR: 51% and 24%	NA
Suzuki et al., 2023 [50]	127 (HFEM/CM:54/73)	24 weeks	P S Japan	Change MMD/MHD and responders at 6 months	Predictors of responder at 6 months	*Primary endpoints:* MMD: −6.9 MHD: −9.7 ≥50%, ≥75%, and 100% responders: HFEM: 90.4, 36.5, and 9.6%; CM: 52.2, 14.9, and 1.5% *Secondary endpoints:* Higher percentages of nausea at baseline were associated with a ≥50% MMD reduction at 6 months.	NA
Caponetto et al., 2023 [51]	83 (HFEM/CM:16/67)	52 weeks	P M (n = 17) Italy	Change MMD, MHD, RR, and persistence in medication overuse at 3–6 and 12 months	Change in MAI, MIDAS, and HIT-6 at 3–6 and 12 months	*Primary endpoints:* MMD: −5, −6, and −6.5 (*p* < 0.001) MHD: −11, −13, and −15 (*p* < 0.001) ≥50%, ≥75%, and 100% responders at 12 months: HFEM: 78.6, 35.7%, and 14.5; CM: 75.9%, 37%, and 56% *Secondary endpoints:* MAI: −6, −7.5, and −7; −14, −15, and −15.5; HIT-6: −11, −20, and −18.5; −11, −12.5, and −15; MIDAS: −18, −18, and −18; −48, −52, and −53.5, respectively, in HFEM and CM.	AEs 9.6% Discontinued for tolerability 1 pts (1.2%) local allergic reaction at site injection, constipation: 7.2%, injecion site reactions: 3.6%.
Barbanti et al., 2024 [6]	130 (HFEM/CM: 49/81)	48 weeks	P M (n = 26) N = Italy	Change in MMD and MHD at weeks 45–48 vs. baseline	Changes in MAI, NRS, HIT-6, MIDAS, and ≥50%, ≥75%, and 100% RR at weeks 45–48 vs. baseline. ≥50%, ≥75%, and 100% RR in patients with psychiatric comorbidities and MO.	*Primary endpoints:* MMD: −6.4(*p* < 0.001) MHD: −14.5 (*p* < 0.001) *Secondary endpoints:* ≥50%, ≥75%, and 100% responders: HFEM: 75.5%, 36.7%, and 2%; CM: 71.6%, 44.4%, and 3.7%; pts with psychiatric comorbidities: 60.5%, 37.2%, and 2.3%; CM with MO: 74.2%, 50%, and 4.8%; CM with MO and psychiatric comorbidities: 60.9%, 39.1%, and 4.3%. NRS: −3.4, −3.4; MAI: −6, −16.5; HIT-6: −16.9, −17.9; MIDAS: −50.4, −76.6, respectively, in HFEM and CM.	TEAEs occurred in 7.8% (6/130) of patients treated with fremanezumab for at least 48 weeks.

**Table 6:** Overview of adverse events among patients receiving fremanezumab in RWE studies.

		AE Type	Frequency Range	Frequency < 8% (pts n)	Frequency 8–10% (pts n)	Frequency > 10% (pts n)
Pts with AEs	0–26.9%	- injection site reactions	1.1–12.7%	6	1	1
		- constipation	0.7–7.2%	7	-	-
		- dizzines	1.9–4%	2	-	-
		- flu-like symptoms	3.9–4-%	2	-	-
		- fatigue	0.4–3%	2	-	-
		- arthralgia	0–2%	1	-	-
		- nausea	0.2–2%	2	-	-
		- abdominal pain	0–1.9%	1	-	-
		- hair loss	0.2–1%	2	-	-
		- libido loss	0–0.2%	1	-	-
Pts with SAEs	0%	-				
Pts who discontinued treatment due to AEs	0–1.2%	- injection site reactions				

AEs: adverse events; SAEs: serious adverse events.

**Table 7:** Galcanezumab in RWE studies.

Author/ Year	N° of pts	Observation Period	Study Type/Center/ National–International	Primary Endpoint	Secondary Endpoint	Results	Safety Findings
Takizawa et al., 2021 [52]	52 pts (EM/CM: 25/27)	12 weeks	R S N = Japan	Change in MMD, MHD, 50% RR, 100% RR. NRS, MAI	Changes from baseline in associated symptoms and premonitory symptoms.	*Primary endpoints:* MMD: −4.4 (*p* < 0.001) in EM. MHD: −7.3 (*p* < 0.001). MAI in EM: −4 (*p* < 0.001). MAI in CM: −6 (*p* < 0.001). The ≥50% and 100% RR: 76.0% and 20.0% in EM and 48.1% and 7.4% in CM. NRS: −2 in EM; −1 in CM (both *p* < 0.001) *Secondary endpoints:* improvement in photophobia, phonophobia, nausea/vomiting 64.9%, 50.0%, and 63.9% respectively. Premonitory symptoms at baseline: 46.1%. After 12 weeks, 62.5% reported premonitory symptoms without subsequent headaches.	Injection site reactions at first, second and third injections: 26.9%, 17.3%, and 20.0%; constipation: 7.7%; fatigue: 5.8%; burning sensation: 3.8%; lightheadedness: 3.8%; others: 19%.
Vernieri et al., 2021 [53]	165 (EM/CM: 33/130)	24 weeks	P M (n = 13) N = Italy	Change in MMD (for HFEM) and MHD (for CM) after 6 months.	Changes in NRS, MAI, HIT-6, and MIDAS scores, ≥50% RR, conversion rate from CM to EM, and MO discontinuation.	*Primary endpoints*: MMD −8 (*p* < 0.001). MHD: −13 (*p* < 0.001). *Secondary endpoints:* NRS: −2 in EM and −2 in CM (*p* < 0.001); HIT-6: −14 in EM and −13 in CM (all *p* < 0.001); MIDAS: −27 in EM and −54 in CM (*p* < 0.001). MAI: −8 in EM; −15 in CM (both *p* < 0.001) ≥50%RRs: 76.5% in HFEM and 63.5% in CM. CM - → EM: 77.2%. MO discontinuation: 82.0%.	AE: 10.3% non-serious events.
Vernieri et al., 2022 [54]	CM:156	12 weeks	P M (n = 14) N = Italy	Consecutive 3-month ≥50% MHD RR.	Persistence of conversion from MO to non-MO and from CM to EM in all 3 months of treatment. Change in MHDs, MIDAS, HIT-6, MAI.	*Primary endpoint:* persistent ≥50% MHD RR: 41.7% *Secondary endpoints:* CM → EM: 55.8%. Conversion from MO to non-MO: 61.8% of patients. MHD: −15 (*p* < 0.001); MIDAS: −43 (*p* < 0.001); HIT-6: −11 (*p* < 0.001); NRS: −2 (*p* < 0.001); MAI: −13 (*p* < 0.001).	NA
Altamura et al., 2022 [55]	CM: 161	52 weeks	P M (n = 15) N = Italy	Conversion rate from CM to EM from baseline to 12 months.	MO discontinuation, changes in MAI, and monthly NRS.	*Primary endpoint:* CM → EM: 52.3%. *Secondary endpoints:* MO discontinuation rate: 82.8%. MAI: −17 (*p* < 0.000001). NRS: −2 (*p* < 0.000001).	NA
Fofi et al., 2022 [56]	27 (EM/CM: 14/13)	24 weeks	P S N = Italy	Change in MMD, NRS, MAI, HIT-6 MIDAS, reduction in RSS, and improvement in MS after 6 months of treatment.	NA	MMD: −10.2 (*p* < 0.001); NRS: −2.4 (*p* < 0.001); MAI: −14.3 (*p* < 0.001); HIT-6: −14.6 (*p* < 0.001); MIDAS: −68.4 (*p* < 0.001); RSS: −7 (*p* = 0.027); MS: +0.29 (*p* = 0.014).	NA
Silvestro et al., 2022 [57]	43 (EM/CM: 8/35)	24 weeks	P S N = Italy	Change in MHD, NRS, attack duration, and whole pain burden score after 3 and 6 months of treatment.	≥50% and ≥75% RR; ≥50% reduction of whole pain burden core. change in MIDAS, HIT-6, MAI MSQ, BDI-II, HDRS scores. Proportion of patients converting from CM to EM and from non-responders to responders to pain killers.	*Primary endpoints:* MHD: −13.1 (T3) and −14.2 (T6) (*p* < 0.001); NRS: −2.1 score (T3) and −2.7 (T6) (*p* < 0.001); Headache attack duration (treated): −5.3 h (T3) and −6.7 h (T6) (*p* < 0.001). Whole total pain burden score: −1498 (T3); −1591.3 (T6) (*p* < 0.001). *Secondary endpoints:* ≥50% RR: 72.1% (T3), 74.4% (T6). ≥75% RR: 44.2% (T3), 55.8% (T6). Reduction of 50% and 75% of the whole total pain burden score: 88.4% (T3), 95.4% (T6), and 76.7% (T3) and 88.4% (T6). MIDAS: −70 (T3) −74.5 (T6) (*p* < 0.001) HIT-6: −11 (T3); −11.5 (T 6) (*p* < 0.001) MAI: −15.5 at moths 6 (*p* < 0.001). MSQ: −45,71 (T3); −47.14 (T6) (*p* < 0.001) BDI-II: −5.5 (T3); −5.5 (T6) (*p* = 0.003) HDRS: −5 (T3); −5 (T6). (*p* < 0.001). CM - → EM: 74.3%	Injection site reaction: 23.26%, constipation:16.27%, fatigue: 6.98%, acrocianosys: 2.32%.
Kwon et al., 2022 [58]	87 (EM/CM:22/65)	12 weeks	P S N = Korea	≥50% RR at 3 months.	≥30%, ≥75%, and 100% RR, MHD, moderate/severe headache days, MAI, CCD, and HIT-6 and MIDAS scores.	*Primary endpoint:* ≥50% RR: 44.8% (54.5% EM and 41.5% CM). *Secondary endpoints:* ≥30% RR: EM 59.1%, CM 55.4%; ≥75% RR: EM 27.3%, CM 27.7%; 100% RR: EM 22.7%, CM 10.8%. MHD: −7.2 (*p* < 0.001). Moderate/severe headache days: −4.3 (*p* < 0.001); MAI: −4.1 (*p* < 0.001); CCD: +7.3 (*p* < 0.001); HIT-6: −4.4 (*p* < 0.001); MIDAS: −32.9 (*p* < 0.001).	Constipation 16.3%, fatigue 7%, acrocyanosis 2.3%
Ashina et al., 2023 [59]	46 (EM/CM: 27/19)	12 weeks	P S N = USA	Effects on premonitory symptoms and/or occurrence of headache afterexposure to triggers or aura episodes in treatment-responders (≥50%), super-responders (≥70%), non-responders (<50%), and super nonresponders(<30%).	NA	Premonitory symptoms decreased by 48% in responders, 28% in non-responders, 50% in super responders, and 12% in super non-responders. Triggers followed by headache decreased by 38% in responders, 13% in non-responders, 31% in super-responders, and 4% in super non-responders.	NA
Guerzoni et al., 2023 [60]	78 CM	64 weeks	P S N = Italy	Change in MMD, MAI after 1 year.	Change in NRS, HIT-6, MIDAS	*Primary endpoints*: MHD: −11.5 (*p* < 0.001); MAI: 30.1 (*p* < 0.001). *Secondary endpoints:* NRS: −2.8; HIT-6: −58.4; MIDAS: −19.5 (*p* < 0.001).	NA
Vernieri et al., 2023 [7]	191 (EM/CM:43/148)	52 weeks	P M (n = 16) N = Italy	Change in MMD /MHD.	Changes in MAI, NRS MIDAS, and HIT-6. ≥50%, ≥75%, and 100% RR.	*Primary endpoints:* MMD: −6.0; MHD: −11.9. (all *p* < 0.00001) Persistent ≥50% RR: 56.5%. Persistent responders have a higher body mass index (BMI) (*p* = 0.007), a good response to triptans (*p* = 0.005), and MMD ≥50% RR at V1 (*p* < 0.0000001). *Secondary endpoints:* EM: MAI –9; NRS: -2; HIT-6: −12.3; MIDAS: −37,6. (*p* < 0.00001). ≥50%, ≥75%, and 100% RR /3.8%, 37.2%, 2.3%. CM: MAI: −18.4, NRS: −1.9; in HIT-6:– 13.7; MIDAS: –57.6. (*p* < 0.00001). ≥30%, ≥75%, and 100% RR. ≥50%, ≥75%, and 100% RR 60.5%, 38.1%, 3.4%.	Two pts dropped out for nonserious AEs.
Suzuki et al., 2023 [61]	55 (EM/CM:18/37)	12 weeks	R S N = Japan	Change in MMD; A ≥ 50% RR at months 1, 2, and 3. WMDs during month 1.	NA	MMD: −6.2 at 1 month (*p* < 0.001), −6.8at 2 months (*p* < 0.001), and −7.9 at 3 months (*p* < 0.001). The≥50% RR: 40.0% at 1 month, 41.8% at 2 months, and 50.9% at 3 months. The≥75% RR: 10.9% at 1 month, 14.5% at 2 months, and 27.3% at 3 months. WMDs week 1: −1.6; week 2: −1.2, week 3: −1.0; week 4: −1.1 (*p* < 0.001).	NA
Lee et al., 2023 [62]	238 CM	12 weeks	P S N = Korea	Change in MHD, a ≥ 50%, ≥ 75%, ≥ 100% RR at month 3. Comparison of migraine characteristics, comorbidities, and treatment responses between responder and non-responder groups.	NA	MHD: −12.7. ≥ 50% RR: 64.3%. ≥ 75% RR: 35.3%. ≥ 100% RR: 3.4%. Responder group features: younger, lower frequency of baseline headache days, more accompanying symptoms such as nausea, vomiting, and photophobia, better triptan response, less depression. Everyday headache (*p* = 0.017), depression (*p* = 0.024), and absence of accompanying symptoms (*p* = 0.020) were significantly associated with response.	NA
Schiano di Cola et al., 2023 [63]	54 (EM/CM:17/37)	24 weeks	R S N = Italy	Change in MHD, MMD, NRS, MAI, MIDAS, and HIT-6 at T0, T3, and T6.	NA	MHD −11.2 at T3 and −11 at T6 (*p* < 0.001). MMD: −8.2 at T3 and −7.5 at T6; (*p* < 0.001). NRS: −1.6 at T3 and −1.7 at T6 (*p* = 0.001). MAI −17.6 at T3 and −17.1 at T6 (*p* < 0.001). MIDAS: −71.8 at T3 and −77.5 at T6 (all *p* < 0.001). HIT-6: −9.3 at 3 M and −11.1 at 6 M (all *p* < 0.001). MIDAS: −74.3% at T3, −80.6% at T6. HIT-6: −24.3% and 29.2% at T3 and T6, respectively.	NA
Schiano di Cola et al., 2023 [64]	47 (EM/CM:17/30)	24 weeks	P S N = Italy	Change in MHD, MMD, NRS, MAI, MIDAS, and HIT-6 scores at T3, and T6.	To evaluate photophobia, photophobia at T3 and T6.	*Primary endpoints:* MHD T3: −10.6; T6: −11.5 (*p* < 0.0001). MMD T3: −7.5; T6: −6.6 (*p* < 0.0001). NRS: −1.4 both T3 and T6 (*p* < 0.0001). MAI: −17.5 at T3 and T6. (*p* < 0.0001). MIDAS T3: −60.5; T6: −64 (*p* < 0.0001). HIT-6 T3: −9; T6: −9.6 (*p* < 0.0001). *Secondary endpoint:* improvement in ictal photophobia: 68.1% more frequent in patients with episodic migraine (*p* = 0.02) and triptans responders (*p* = 0.03).	NA
Kim et al., 2023 [65]	104 (EM/CM: 24/80)	12 weeks	R S N = Korea	The ≥50% RR in the 3rd month of treatment vs. baseline.	NA	The ≥ 50% RR: 55.7%.	NA

**Table 8:** Overview of adverse events among patients receiving galcanezumab in RWE studies.

		AE Type	Frequency Range	Frequency < 8% (pts n)	Frequency 8–10% (pts n)	Frequency > 10% (pts n)
Pts with AEs	0–26.9%	- injection site reaction	1%/26.9%	1	-	2
		- constipation	1.1%/16.3%	2	-	2
		- dizziness	<2%/8%	1	1	-
		- flu-like symptoms	0%/<2%	2	-	-
		- fatigue	5.8%/7%	3	-	-
		- arthralgia	0%/2.3%	2	-	-
		- acrocyanosis	0%/2.3%	2	-	-
Pts with SAEs	0%	-				
Pts who discontinued treatment due to AEs	0	-				

**Table 9:** Multiple mAbs in RWE studies.

Author/ Year/Country	N° of pts	Observation Period	Study Type/Center/ National–International	Primary Endpoint	Secondary Endpoint	Results	Safety Findings
Caronna et al., 2021 [8]	CM: 139 Erenumab: 96, Galcanezumab: 43	24 weeks	P S N = Italy	Change in MHD in MO and non-MO patients at month 6; ≥50% RR at month 6.	To compare pts with and without MO resolution at 6 months.	*Primary endpoint:* MHD MO: −13.4 (*p* <0.0001) MHD non-MO: −7.8 (*p* <0.0001) ≥50% RR MO: 63.6% ≥50% RR non-MO: 57.5% *Secondary endpoint:* pts with ongoing MO at 6 months: higher frequency of MDM (*p* = 0.020), higher score in a 0–3 pain severity scale (*p* = 0.020), higer MAI (*p* =0.049), benzodiazepine use (*p* = 0.034), more anxiety in BAI (*p* =0.020); previous failure to onabotulinumtoxinA (*p* =0.048).	NA
Vernieri et al., 2021 [9]	154 Erenumab: 91, Galcanezumab: 63 (EM/CM: 47/107)	52 weeks 8-weeks follow-up after treatment completion	P M N = Italy	Change in MMD in the three months following erenumab and galcanezumab discontinuation (F-UP 1–2-3) after one year of treatment vs. baseline.	Changes in MAI, in NRS, and HIT-6 score in F-UP 1–2-3.	*Primary endpoint:* MMD F-UP 1 EM/CM: −2/−5.5 MMD F-UP 2: EM/CM: −0.5/−4 MMD F-UP 3: EM/CM: −0.5/−4 *Secondary endpoints:* MAI at F-UP 1 EM/CM: −6/−13 MAI at F-UP 2: EM/CM: −3/−9 MAI at F-UP 3: EM/CM: −2/−6 NRS at F-UP 1 EM/CM: −1.5/−1 NRS at F-UP 2: EM/CM: −1.5/−1 NRS at F-UP 3: EM/CM: −0.5/0 HIT-6 at F-UP 1 EM/CM: −10−5/−7 HIT-6 at F-UP 2: EM/CM: −4/−5 HIT-6 at F-UP 3: EM/CM: −6/−4	NA
Raffaelli et al., 2022 [10]	39 Erenumab: 16, Galcanezuma: 15, Fremanezumab: 8 (HFEM/CM: 14/25)	60 weeks	P S N = Germany	Change in MMD between the last four weeks of treatment discontinuation and weeks 9–12 after restart.	Changes in MHD, MAI, and HIT-6 scores in the same observation period.	*Primary endpoint:* MMD: −4.5 (*p* < 0.001). *Secondary endpoint:* MHD −5.4 (*p* < 0.001) MAI: −3.9 HIT-6: −6 (*p* < 0.001).	NA
Iannone et al., 2022 [11]	CM:203 Erenumab: 96 Galcanezumab: 74 Fremanezumab: 33	52 weeks	P S N = Italy	Change in MMD; the ≥50%, ≥75%, and 100% RR in MMD at 12 months. The ≥50% reduction in MIDAS score at 12 months.	Clinical predictors of response at 6 months and 12 months.	*Primary endopoint:* MMD: −8.4 (*p*< 0.0001) ≥50% RR: 36.4% ≥75% RR: 15.4% 100% RR: not achieved. Reduction ≥ 50% in MIDAS: from 63.5% to 96.1%. *Secondary endpoint:* association with lower RR at 1 month: duration of chronicization (*p* = 0.04); elevated number of MMD at baseline (*p* < 0.0001); association with lower RR at 6 months: duration of chronicization (P = 0.04); MMD at baseline (*p* < 0.0001); Total Number of Analgesics (*p* = 0.003).	NA
Nowaczewska et al., 2022 [12]	123 Erenumab: 75, Fremanezumab: 48 (HFEM/CM:36/87)	12 weeks	R S N = Poland	Check if baseline clinical parameters and cerebral blood flow (CBF) measured by transcranial Doppler (TCD) may help predict mAbs efficacy.	NA	Baseline Vm (mean velocity) values in the middle cerebral artery were significantly lower in good responders vs. non-responders. MAbs responsiveness ≥50% was positively associated with unilateral pain localization (*p* = 0.003) and HIT-6 score (*p* = 0.036), whereas negatively associated with Vm in right MCA (*p* = 0.012) and having no relatives with migraine (*p* = 0.040).	NA
Quintana et al., 2022 [13]	123 (56 Erenumab: 56, Galcanezumab: 38, Fremanezumab: 29 (HFEM/CM:66/57)	24 weeks	R S N = Italy	Reduction in MDM and MAI at 3 and 6 months.	Change in HIT-6, MIDAS, and headwork	*Primary endpoint:* At 3 months, fremanezumab was statistically superior to erenumab (MMD −16.7 vs. −12.9, *p* < 0.02). *Secondary endpoint:* Erenumab determined a greater improvement in the headwork vs. fremanezumab (−14.7 vs. −8.2, *p* < 0.01)	NA
Barbanti et al., 2022 [14]	864 Erenumab: 639, Galcanezumab: 173, Fremanezumab: 52. (HFEM/CM: 208/565)	≥24 weeks	P M (n = 20) N = Italy	≥ 50% response predictors at 24 weeks.	≥ 75% and 100% response predictors at 24 weeks.	*Primary endpoint:* ≥50% response in HFEM positively associated UP + UAs (*p* = 0.004) and in CM with UAs (*p* = 0.0264), UP + UAs (*p* = 0.012), UP + allodynia (*p* = 0.034) *Secondary endpoint:* 75% response positively associated with UP + UAs (*p* = 0.006) in HFEM and with UP + UAs (*p* = 0.012) and UP + allodynia (*p* = 0.005) in CM	30% of erenumab and fremanezumab patients reported TEAEs (pain and redness at the injection site and constipation)
Varnado et al., 2022 [15]	3082 CGRP mAb versus SOC and 421 Galcanezumab versus SOC EM/CM 1749/ 1333	52 weeks	R S N = USA	To compare real-world treatment patterns for CGRP mAb, specifically galcanezumab versus standard-of-care (SOC) migraine preventive treatments.	NA	Pts stopping SOC: 75%. Compared with SOC, the CGRP mAb cohort had higher mean persistence (212.5 vs. 131.9 days), adherence (PDC: 55.1% vs. 35.2%), and more patients were adherent with PDC ≥80% (32.7% vs. 18.7%) (all *p* < 0.001). During 12-month follow-up, fewer patients discontinued CGRP mAb versus SOC (58.8% vs. 77.6%, *p* < 0.001).	NA
Katsuki et al., 2023 [16]	8 CM Fremanezumab: 5; Galcanezumab: 3	12 weeks	R S N = Japan	Median of MHD, MAI HIT-6 at 3 months.	NA	Median MHD before, one, and three months: 30, 4 and 1, respectively. Median MAI: 17.5, 1.5, and 0, respectively) Median for HIT-6: 60.5, 45.5, and 44, respectively.	NA
Cantarelli et al., 2023 [17]	104 CM Erenumab: 48, Galcanezumab: 43, Fremanezumab: 13	24 weeks	R S N = Spain	Change in MHD, MIDAS, and HIT-6 at weeks 0, 12, and 24 of treatment (at least 50%)	Treatment efficacy: young versus older patients, previous failure to >5 versus <5 drugs.	*Primary endpoint:* Reduction from erenumab, galcanezumab, and fremanezumab in MHD, MIDAS, and HIT-6 at week 12 (*p* < 0.001); MHD at week 24 from Erenumab and Galcanezumab (*p* <0.001); MIDAS and HIT-6 in the erenumab group (*p* < 0.001, *p* = 0.004) and MIDAS in the galcanezumab group (*p* < 0.001). *Secondary endpoints:* Reduction in MHD ≥50% at week 12 (*p* = 0.044) was observed between patients with >5 prior treatment failures with fremanezumab and in MHD ≥ 75% at week 24 with galcanezumab (*p* = 0.038).	NA
Muñoz-Vendrell et al., 2023 [18]	162 Erenumab: 38, Galcanezumab: 85, Fremanezumab: 29 (HFEM/CM: 32/130)	24 weeks	P M (n = 18) N = Spain	Change in MMD at 6 months of treatment and the presence of AEs	Change in MMD at 3 months, change in MHD, MAI, frequency of days by intensity, the 30%, 50%, 75%, and 100% RR, HIT-6, MIDAS, and PGIC at 3 and 6 months.	*Primary endpoint:* MMD: −10.1 (*p* = 0.0001). *Secondary endpoint:* MMD: −9.7; MHD: −10.1, −10.5; MAI: −8.8, −9.4 (*p* < 0.001). ≥30%, ≥50%, ≥75%, and 100% RR: 68%, 57%, 33%, and 9%.	Injection pain, rash, or pruritus: 26 pts, flu-like symptoms: 8 pts, and hair loss: 2 pts.
Guerzoni et al., 2023 [19]	233 Erenumab, Galcanezumab, Fremanezumab (HFEM/CM:40/193)	48 weeks	P S N = Italy	Response to anti-CGRP mAbs between women in menopause and those of childbearing age.	Effectiveness of anti-CGRP mAbs between women with physiological menopause and those with a surgical one and effectiveness of different antibodies and the predictors of a 75% response among women in menopause.	*Primary endpoint:* The effectiveness of anti-CGRP monoclonal antibodies is almost the same between women in menopause and women of childbearing age. *Secondary endpoint:* No predictors of an excellent response apart from a lower AC at the baseline (*p* = 0.03).	Constipation: erenumab 54.5% for galcanezumab: 40.9%; fremanezumab, 4.5%. Injection site reaction only in 7 pts with galcanezumab.
Barbanti et al., 2023 [20]	771 Erenumab: 527, Fremanezumab: 40, Galcanezumab:5 (HFEM/CM:154/418)	≥24 weeks	P M (n = 16) N = Italy	Frequency and characteristics of late responders (≥12 weeks)	NA	Late responders: 55.1%. Differed from responders: higher BMI (+0.78, *p* = 0.024), more frequent treatment failures (+0.52, *p* = 0.017) and psychiatric comorbidities (+10.1%, *p* = 0.041), and less common unilateral pain, alone (−10,9%, *p* = 0.025) or in combination with UAs (−12.3%, *p* = 0.006) or allodynia (−10.7, *p* = 0.01).	AEs: 23% constipation (4.4%), fatigue (4.4%), and dizziness (3.3%).
Vernieri et al., 2023 [21]	226 Erenumab: 125, Galcanezumab, Fremanezumab: 101 (HFEM/CM:46/180)	64 weeks	P M (n = 10) N = Italy	MMD, MAI, and HIT-6 at baseline, after 90–112 days (Rev-1), after 84–90 days since Rev-1 (Rev-2) and 30 days after the last injection, in the first and second year after a discontinuation period.	NA	MMD (18.1 ± 7.8 vs. 3.4 ± 7.8), MAI (26.7± 28.3 vs.17.7 ±17.2), and HIT-6 scores (63.1 ± 5.9 vs. 67.1 ± 10.3) were lower in the second year than in the pre-treatment baseline (consistently, *p* < 0.0001). Second-year baseline MMD were lower in patients on anti-CGRP mAbs (*p* = 0.001) and with lower pre-treatment baseline MMD (*p* ≤ 0.001).	NA

AE: adverse event AMD: monthly acute medication intake days; BAI: Beck Anxiety Inventory scale; BDI-II—Beck Depression Inventory, Second Edition; BMI: body mass index; CCD: clear crystal days; CM: chronic migraine; EM: episodic migraine; HADS: Hospital Anxiety and Depression Scale; HARS—Hamilton Anxiety Rating Scale; HFEM: high-frequency episodic migraine; HIT-6: Headache Impact Test; LFEM: low-frequency episodic migraine; MAI: monthly acute medication intake; MIDAS: Migraine Disability Assessment Questionnaire; MMD:m migraine days; MOH: medication overuse–headache; MSQ: Migraine-Specific Quality-of-Life Questionnaire; NA: not applicable; NRS: Numerical Rating Scale; PGIC: Patient Global Impression of Change scale; PMI: monthly painkiller intake; PDC: proportion of days covered; RR: response rate; SOC: standard of care; UMP: unilateral migraine pain; WMD: weekly migraine days.

## Data Availability

The data used for this review consist of published articles and are available on PubMed. The primary sources are publicly accessible through PubMed at https://pubmed.ncbi.nlm.nih.gov (accessed on 22 August 2024) using the respective DOIs or PMIDs provided in the bibliography.

## References

[1] Haghdoost F., Puledda F., Garcia-Azorin D., Huessler E.-M., Messina R., Pozo-Rosich P. (2023). Evaluating the efficacy of CGRP mAbs and gepants for the preventive treatment of migraine: A systematic review and network meta-analysis of phase 3 randomised controlled trials. Cephalalgia Int. J. Headache.

[2] Serra López-Matencio J.M., Gago-Veiga A.B., Gómez M., Alañón Plaza E., Mejía G.P., González-Gay M.Á., Castañeda S. (2022). Treatment of migraine with monoclonal antibodies. Expert Opin. Biol. Ther..

[3] Blonde L., Khunti K., Harris S.B., Meizinger C., Skolnik N.S. (2018). Interpretation and Impact of Real-World Clinical Data for the Practicing Clinician. Adv. Ther..

[4] Barbanti P., Aurilia C., Cevoli S., Egeo G., Fofi L., Messina R., Salerno A., Torelli P., Albanese M., Carnevale A. (2021). Long-term (48 weeks) effectiveness, safety, and tolerability of erenumab in the prevention of high-frequency episodic and chronic migraine in a real world: Results of the EARLY 2 study. Headache.

[5] Barbanti P., Egeo G., Aurilia C., Altamura C., d’Onofrio F., Finocchi C., Albanese M., Aguggia M., Rao R., Zucco M. (2022). Predictors of response to anti-CGRP monoclonal antibodies: A 24-week, multicenter, prospective study on 864 migraine patients. J. Headache Pain.

[6] Barbanti P., Egeo G., Proietti S., d’Onofrio F., Aurilia C., Finocchi C., Di Clemente L., Zucco M., Doretti A., Messina S. (2024). Assessing the Long-Term (48-Week) Effectiveness, Safety, and Tolerability of Fremanezumab in Migraine in Real Life: Insights from the Multicenter, Prospective, FRIEND3 Study. Neurol. Ther..

[7] Vernieri F., Brunelli N., Marcosano M., Aurilia C., Egeo G., Lovati C., Favoni V., Perrotta A., Maestrini I., Rao R. (2023). Maintenance of response and predictive factors of 1-year GalcanezumAb treatment in real-life migraine patients in Italy: The multicenter prospective cohort GARLIT study. Eur. J. Neurol..

[8] Caronna E., Gallardo V.J., Alpuente A., Torres-Ferrus M., Pozo-Rosich P. (2021). Anti-CGRP monoclonal antibodies in chronic migraine with medication overuse: Real-life effectiveness and predictors of response at 6 months. J. Headache Pain.

[9] Vernieri F., Brunelli N., Messina R., Costa C.M., Colombo B., Torelli P., Quintana S., Cevoli S., Favoni V., d’Onofrio F. (2021). Discontinuing monoclonal antibodies targeting CGRP pathway after one-year treatment: An observational longitudinal cohort study. J. Headache Pain.

[10] Raffaelli B., Terhart M., Mecklenburg J., Neeb L., Overeem L.H., Siebert A., Steinicke M., Reuter U. (2022). Resumption of migraine preventive treatment with CGRP(-receptor) antibodies after a 3-month drug holiday: A real-world experience. J. Headache Pain.

[11] Iannone L.F., Fattori D., Benemei S., Chiarugi A., Geppetti P., De Cesaris F. (2022). Long-Term Effectiveness of Three Anti-CGRP Monoclonal Antibodies in Resistant Chronic Migraine Patients Based on the MIDAS score. CNS Drugs.

[12] Nowaczewska M., Straburzyński M., Waliszewska-Prosół M., Meder G., Janiak-Kiszka J., Kaźmierczak W. (2022). Cerebral Blood Flow and Other Predictors of Responsiveness to Erenumab and Fremanezumab in Migraine—A Real-Life Study. Front. Neurol..

[13] Quintana S., Russo M., Manzoni G.C., Torelli P. (2022). Comparison study between erenumab, fremanezumab, and galcanezumab in the preventive treatment of high frequency episodic migraine and chronic migraine. Neurol. Sci. Off. J. Ital. Neurol. Soc. Ital. Soc. Clin. Neurophysiol..

[14] Barbanti P., Aurilia C., Egeo G., Torelli P., Proietti S., Cevoli S., Bonassi S. (2023). Late Response to Anti-CGRP Monoclonal Antibodies in Migraine: A Multicenter Prospective Observational Study. Neurology.

[15] Varnado O.J., Manjelievskaia J., Ye W., Perry A., Schuh K., Wenzel R. (2022). Treatment Patterns for Calcitonin Gene-Related Peptide Monoclonal Antibodies Including Galcanezumab versus Conventional Preventive Treatments for Migraine: A Retrospective US Claims Study. Patient Prefer. Adherence.

[16] Katsuki M., Kashiwagi K., Kawamura S., Tachikawa S., Koh A. (2023). One-Time Use of Galcanezumab or Fremanezumab for Migraine Prevention. Cureus.

[17] Cantarelli L., Pestana Grafiña D., Gonzalez Perez A., García Gil S., Gutiérrez Nicolás F., Ramos Santana E., Navarro Dávila M.A., Otazo Pérez S.M., Calzado Gómez G., Perez Reyes S. (2023). Efficacy and Safety of Erenumab, Galcanezumab, and Fremanezumab in the Treatment of Drug-Resistant Chronic Migraine: Experience in Real Clinical Practice. Ann. Pharmacother..

[18] Muñoz-Vendrell A., Campoy S., Caronna E., Alpuente A., Torres-Ferrus M., Nieves Castellanos C., Olivier M., Campdelacreu J., Prat J., Camiña Muñiz J. (2023). Effectiveness and safety of anti-CGRP monoclonal antibodies in patients over 65 years: A real-life multicentre analysis of 162 patients. J. Headache Pain.

[19] Guerzoni S., Castro F.L., Brovia D., Baraldi C., Pani L. (2024). Evaluation of the risk of hypertension in patients treated with anti-CGRP monoclonal antibodies in a real-life study. Neurol. Sci. Off. J. Ital. Neurol. Soc. Ital. Soc. Clin. Neurophysiol..

[20] Barbanti P., Aurilia C., Egeo G., Proietti S., D’Onofrio F., Torelli P., Aguggia M., Bertuzzo D., Finocchi C., Trimboli M. (2024). Ultra-late response (>24 weeks) to anti-CGRP monoclonal antibodies in migraine: A multicenter, prospective, observational study. J. Neurol..

[21] Vernieri F., Brunelli N., Guerzoni S., Iannone L.F., Baraldi C., Rao R., Schiano di Cola F., Ornello R., Cevoli S., Lovati C. (2023). Retreating migraine patients in the second year with monoclonal antibodies anti-CGRP pathway: The multicenter prospective cohort RE-DO study. J. Neurol..

[22] Barbanti P., Aurilia C., Egeo G., Fofi L. (2019). Erenumab: From scientific evidence to clinical practice-the first Italian real-life data. Neurol. Sci. Off. J. Ital. Neurol. Soc. Ital. Soc. Clin. Neurophysiol..

[23] Barbanti P., Aurilia C., Egeo G., Fofi L., Cevoli S., Colombo B., Filippi M., Frediani F., Bono F., Grazzi L. (2021). Erenumab in the prevention of high-frequency episodic and chronic migraine: Erenumab in Real Life in Italy (EARLY), the first Italian multicenter, prospective real-life study. Headache J. Head Face Pain.

[24] Scheffler A., Messel O., Wurthmann S., Nsaka M., Kleinschnitz C., Glas M., Naegel S., Holle D. (2020). Erenumab in highly therapy-refractory migraine patients: First German real-world evidence. J. Headache Pain.

[25] Ornello R., Casalena A., Frattale I., Gabriele A., Affaitati G., Giamberardino M.A., Assetta M., Maddestra M., Marzoli F., Viola S. (2020). Real-life data on the efficacy and safety of erenumab in the Abruzzo region, central Italy. J. Headache Pain.

[26] Russo A., Silvestro M., Scotto di Clemente F., Trojsi F., Bisecco A., Bonavita S., Tessitore A., Tedeschi G. (2020). Multidimensional assessment of the effects of erenumab in chronic migraine patients with previous unsuccessful preventive treatments: A comprehensive real-world experience. J. Headache Pain.

[27] Lambru G., Hill B., Murphy M., Tylova I., Andreou A.P. (2020). A prospective real-world analysis of erenumab in refractory chronic migraine. J. Headache Pain.

[28] Ornello R., Baraldi C., Guerzoni S., Lambru G., Fuccaro M., Raffaelli B., Gendolla A., Barbanti P., Aurilia C., Cevoli S. (2021). Gender Differences in 3-Month Outcomes of Erenumab Treatment-Study on Efficacy and Safety of Treatment With Erenumab in Men. Front. Neurol..

[29] de Vries Lentsch S., Verhagen I.E., van den Hoek T.C., Maassen Van DenBrink A., Terwindt G.M. (2021). Treatment with the monoclonal calcitonin gene-related peptide receptor antibody erenumab: A real-life study. Eur. J. Neurol..

[30] De Matteis E., Affaitati G., Frattale I., Caponnetto V., Pistoia F., Giamberardino M.A., Sacco S., Ornello R. (2021). Early outcomes of migraine after erenumab discontinuation: Data from a real-life setting. Neurol. Sci. Off. J. Ital. Neurol. Soc. Ital. Soc. Clin. Neurophysiol..

[31] Faust E., Pivneva I., Yang K., Betts K.A., Ahmed Z., Joshi S., Hogan R., Blumenfeld A., Schim J., Feoktistov A. (2021). Real-World Treatment Profiles, Clinical Outcomes, and Healthcare Resource Utilization of Patients with Migraine Prescribed Erenumab: A Multicenter Chart-Review Study of US Headache Centers. Neurol. Ther..

[32] Andreou A.P., Fuccaro M., Hill B., Murphy M., Caponnetto V., Kilner R., Lambru G. (2022). Two-year effectiveness of erenumab in resistant chronic migraine: A prospective real-world analysis. J. Headache Pain.

[33] Becker W.J., Spacey S., Leroux E., Giammarco R., Gladstone J., Christie S., Akaberi A., Power G.S., Minhas J.K., Mancini J. (2022). A real-world, observational study of erenumab for migraine prevention in Canadian patients. Headache J. Head Face Pain.

[34] Pensato U., Favoni V., Pascazio A., Benini M., Asioli G.M., Merli E., Calabrò C., Cortelli P., Pierangeli G., Cevoli S. (2020). Erenumab efficacy in highly resistant chronic migraine: A real-life study. Neurol. Sci. Off. J. Ital. Neurol. Soc. Ital. Soc. Clin. Neurophysiol..

[35] Cullum C.K., Do T.P., Ashina M., Bendtsen L., Hugger S.S., Iljazi A., Gusatovic J., Snellman J., Lopez-Lopez C., Ashina H. (2022). Real-world long-term efficacy and safety of erenumab in adults with chronic migraine: A 52-week, single-center, prospective, observational study. J. Headache Pain.

[36] Khalil M., Moreno-Ajona D., Villar-Martínez M.D., Greenwood F., Hoffmann J., Goadsby P.J. (2022). Erenumab in chronic migraine: Experience from a UK tertiary centre and comparison with other real-world evidence. Eur. J. Neurol..

[37] Alsaadi T., Noori S., Varakian R., Youssef S., Almadani A. (2022). Real-world experience of erenumab in patients with chronic or episodic migraine in the UAE. BMC Neurol..

[38] Ornello R., Baraldi C., Guerzoni S., Lambru G., Andreou A.P., Raffaelli B., Gendolla A., Barbanti P., Aurilia C., Egeo G. (2022). Comparing the relative and absolute effect of erenumab: Is a 50% response enough? Results from the ESTEEMen study. J. Headache Pain.

[39] Gantenbein A.R., Agosti R., Kamm C.P., Landmann G., Meier N., Merki-Feld G.S., Petersen J.A., Pohl H., Ryvlin P., Schankin C.J. (2022). Swiss QUality of life and healthcare impact Assessment in a Real-world Erenumab treated migraine population (SQUARE study): Interim results. J. Headache Pain.

[40] Cetta I., Messina R., Zanandrea L., Colombo B., Filippi M. (2022). Comparison of efficacy and safety of erenumab between over and under 65-year-old refractory migraine patients: A pivotal study. Neurol. Sci. Off. J. Ital. Neurol. Soc. Ital. Soc. Clin. Neurophysiol..

[41] Lanteri-Minet M., Fabre R., Martin C., Pradat K., Alchaar A., Bozzolo E., Duchene M.L., Van Obberghen E.K., Donnet A., Fontaine D. (2023). One-year prospective real-world assessment of effectiveness and safety of erenumab in migraine prevention: Results of the French FHU INOVPAIN registry study. J. Headache Pain.

[42] Troy E., Shrukalla A.A., Buture A., Conaty K., Macken E., Lonergan R., Melling J., Long N., Shaikh E., Birrane K. (2023). Medium-term real-world data for erenumab in 177 treatment resistant or difficult to treat chronic migraine patients: Persistence and patient reported outcome measures after 17–30 months. J. Headache Pain.

[43] Pilati L., Torrente A., Di Marco S., Ferlisi S., Notaro G., Romano M., Alonge P., Vassallo L., Ferraù L., Autunno M. (2023). Erenumab and Possible CGRP Effect on Chronotype in Chronic Migraine: A Real-Life Study of 12 Months Treatment. J. Clin. Med..

[44] Buture A., Tomkins E.M., Shukralla A., Troy E., Conaty K., Macken E., Lonergan R., Melling J., Long N., Birrane K. (2023). Two-year, real-world erenumab persistence and quality of life data in 82 pooled patients with abrupt onset, unremitting, treatment refractory headache and a migraine phenotype: New daily persistent headache or persistent post-traumatic headache in the majority of cases. Cephalalgia Int. J. Headache.

[45] Barbanti P., Egeo G., Aurilia C., d’Onofrio F., Albanese M., Cetta I., Di Fiore P., Zucco M., Filippi M., Bono F. (2022). Fremanezumab in the prevention of high-frequency episodic and chronic migraine: A 12-week, multicenter, real-life, cohort study (the FRIEND study). J. Headache Pain.

[46] Driessen M.T., Cohen J.M., Thompson S.F., Patterson-Lomba O., Seminerio M.J., Carr K., Totev T.I., Sun R., Yim E., Mu F. (2022). Real-world effectiveness after initiating fremanezumab treatment in US patients with episodic and chronic migraine or difficult-to-treat migraine. J. Headache Pain.

[47] Barbanti P., Egeo G., Aurilia C., Torelli P., Finocchi C., d’Onofrio F., d’Onofrio L., Rao R., Messina S., Di Clemente L. (2023). Early and sustained efficacy of fremanezumab over 24-weeks in migraine patients with multiple preventive treatment failures: The multicenter, prospective, real-life FRIEND2 study. J. Headache Pain.

[48] Argyriou A.A., Dermitzakis E.V., Xiromerisiou G., Rallis D., Soldatos P., Litsardopoulos P., Vikelis M. (2023). Efficacy and safety of fremanezumab for migraine prophylaxis in patients with at least three previous preventive failures: Prospective, multicenter, real-world data from a Greek registry. Eur. J. Neurol..

[49] Cullum C.K., Chaudhry B.A., Do T.P., Amin F.M. (2023). Real-world efficacy and tolerability of fremanezumab in adults with chronic migraine: A 3-month, single-center, prospective, observational study. Front. Neurol..

[50] Suzuki S., Suzuki K., Shiina T., Haruyama Y., Hirata K. (2023). Real-world experience with monthly and quarterly dosing of fremanezumab for the treatment of patients with migraine in Japan. Front. Neurol..

[51] Caponnetto V., Russo A., Silvestro M., Tessitore A., De Icco R., Vaghi G., Sances G., Tassorelli C., Baraldi C., Castro F.L. (2023). Long-Term Treatment Over 52 Weeks with Monthly Fremanezumab in Drug-Resistant Migraine: A Prospective Multicenter Cohort Study. CNS Drugs.

[52] Takizawa T., Ohtani S., Watanabe N., Miyazaki N., Ishizuchi K., Sekiguchi K., Iba C., Shibata M., Takemura R., Hori S. (2022). Real-world evidence of galcanezumab for migraine treatment in Japan: A retrospective analysis. BMC Neurol..

[53] Vernieri F., Altamura C., Brunelli N., Costa C.M., Aurilia C., Egeo G., Fofi L., Favoni V., Lovati C., Bertuzzo D. (2022). Rapid response to galcanezumab and predictive factors in chronic migraine patients: A 3-month observational, longitudinal, cohort, multicenter, Italian real-life study. Eur. J. Neurol..

[54] Vernieri F., Altamura C., Brunelli N., Costa C.M., Aurilia C., Egeo G., Fofi L., Favoni V., Pierangeli G., Lovati C. (2021). Galcanezumab for the prevention of high frequency episodic and chronic migraine in real life in Italy: A multicenter prospective cohort study (the GARLIT study). J. Headache Pain.

[55] Altamura C., Brunelli N., Marcosano M., Aurilia C., Egeo G., Lovati C., Favoni V., Perrotta A., Maestrini I., Schiano Di Cola F. (2022). Conversion from chronic to episodic migraine in patients treated with galcanezumab in real life in Italy: The 12-month observational, longitudinal, cohort multicenter GARLIT experience. J. Neurol..

[56] Fofi L., Altamura C., Fiorentini G., Brunelli N., Marcosano M., Barbanti P., Vernieri F. (2022). Improving distress perception and mutuality in migraine caregivers after 6 months of galcanezumab treatment. Headache.

[57] Silvestro M., Tessitore A., Orologio I., De Micco R., Tartaglione L., Trojsi F., Tedeschi G., Russo A. (2022). Galcanezumab effect on “whole pain burden” and multidimensional outcomes in migraine patients with previous unsuccessful treatments: A real-world experience. J. Headache Pain.

[58] Kwon S., Gil Y.-E., Lee M.J. (2022). Real-world efficacy of galcanezumab for the treatment of migraine in Korean patients. Cephalalgia.

[59] Ashina S., Melo-Carrillo A., Toluwanimi A., Bolo N., Szabo E., Borsook D., Burstein R. (2023). Galcanezumab effects on incidence of headache after occurrence of triggers, premonitory symptoms, and aura in responders, non-responders, super-responders, and super non-responders. J. Headache Pain.

[60] Guerzoni S., Baraldi C., Castro F.L., Cainazzo M.M., Pani L. (2023). Galcanezumab for the treatment of chronic migraine and medication overuse headache: Real-world clinical evidence in a severely impaired patient population. Brain Behav..

[61] Suzuki K., Suzuki S., Shiina T., Haruyama Y., Fujita H., Funakoshi K., Hirata K. (2023). Could efficacy at 1 week after galcanezumab administration for patients with migraine predict responders at 3 months? A real world study. J. Neurol..

[62] Lee H.C., Cho S., Kim B.-K. (2023). Predictors of response to galcanezumab in patients with chronic migraine: A real-world prospective observational study. Neurol. Sci. Off. J. Ital. Neurol. Soc. Ital. Soc. Clin. Neurophysiol..

[63] Schiano di Cola F., Ceccardi G., Bolchini M., Caratozzolo S., Liberini P., Padovani A., Rao R. (2023). Migraine Disability Improvement during Treatment with Galcanezumab in Patients with Chronic and High Frequency Episodic Migraine. Neurol. Int..

[64] Schiano di Cola F., Ceccardi G., Bolchini M., Caratozzolo S., Liberini P., Padovani A., Rao R. (2023). Photophobia and migraine outcome during treatment with galcanezumab. Front. Neurol..

[65] Kim S.A., Jang H., Lee M.J. (2023). Predictors of galcanezumab response in a real-world study of Korean patients with migraine. Sci. Rep..

[66] Sacco S., Lampl C., Amin F.M., Braschinsky M., Deligianni C., Uludüz D., Versijpt J., Ducros A., Gil-Gouveia R., Katsarava Z. (2022). European Headache Federation (EHF) consensus on the definition of effective treatment of a migraine attack and of triptan failure. J. Headache Pain.

